# MUT-7 exoribonuclease activity and localization are mediated by an ancient domain

**DOI:** 10.1093/nar/gkae610

**Published:** 2024-07-23

**Authors:** Virginia Busetto, Lizaveta Pshanichnaya, Raffael Lichtenberger, Stephan Hann, René F Ketting, Sebastian Falk

**Affiliations:** Max Perutz Labs, Vienna Biocenter Campus (VBC), Dr.-Bohr-Gasse 9, 1030 Vienna, Austria; Department of Structural and Computational Biology, Max Perutz Labs, University of Vienna, Campus Vienna Biocenter 5, 1030 Vienna, Austria; Biology of Non-coding RNA Group, Institute of Molecular Biology, Ackermannweg 4, 55128 Mainz, Germany; International PhD Programme on Gene Regulation, Epigenetics & Genome Stability, Mainz, Germany; Max Perutz Labs, Vienna Biocenter Campus (VBC), Dr.-Bohr-Gasse 9, 1030 Vienna, Austria; Department of Structural and Computational Biology, Max Perutz Labs, University of Vienna, Campus Vienna Biocenter 5, 1030 Vienna, Austria; Institute of Analytical Chemistry, Department of Chemistry, University of Natural Resources and Life Sciences, Vienna, Muthgasse 18, 1190 Vienna, Austria; Biology of Non-coding RNA Group, Institute of Molecular Biology, Ackermannweg 4, 55128 Mainz, Germany; Institute of Developmental Biology and Neurobiology, Johannes Gutenberg University, 55099Mainz, Germany; Max Perutz Labs, Vienna Biocenter Campus (VBC), Dr.-Bohr-Gasse 9, 1030 Vienna, Austria; Department of Structural and Computational Biology, Max Perutz Labs, University of Vienna, Campus Vienna Biocenter 5, 1030 Vienna, Austria

## Abstract

The MUT-7 family of 3′–5′ exoribonucleases is evolutionarily conserved across the animal kingdom and plays essential roles in small RNA production in the germline. Most MUT-7 homologues carry a C-terminal domain of unknown function named MUT7-C appended to the exoribonuclease domain. Our analysis shows that the MUT7-C is evolutionary ancient, as a minimal version of the domain exists as an individual protein in prokaryotes. In animals, MUT7-C has acquired an insertion that diverged during evolution, expanding its functions. *Caenorhabditis elegans* MUT-7 contains a specific insertion within MUT7-C, which allows binding to MUT-8 and, consequently, MUT-7 recruitment to germ granules. In addition, in *C. elegans* and human MUT-7, the MUT7-C domain contributes to RNA binding and is thereby crucial for ribonuclease activity. This RNA-binding function most likely represents the ancestral function of the MUT7-C domain. Overall, this study sheds light on MUT7-C and assigns two functions to this previously uncharacterized domain.

## Graphical Abstract

**Graphical Abstract:**
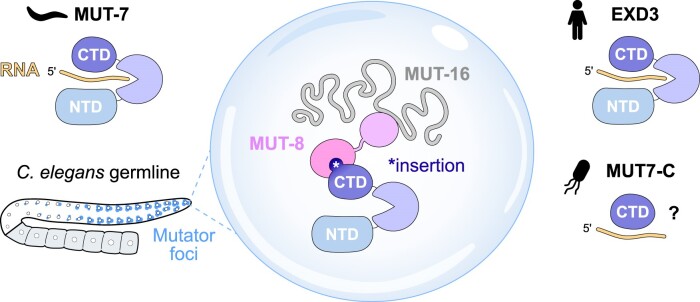

## Introduction

Animals employ RNA interference (RNAi) pathways as a defence mechanism against transposable elements (TEs) and other non-self elements in the germline (1,2). This ensures genome integrity and allows the faithful transmission of genetic information to future generations. RNAi is centred around Argonaute (AGO) proteins binding small RNAs (sRNAs) to form the RNA-induced silencing complex (RISC), which recognizes by sequence complementarity the target RNA of the non-self elements (3). The recognition of the target RNA can trigger transcriptional or post-transcriptional silencing pathways, resulting in gene repression (1,2). To generate an effective RNAi response, animals use different amplification strategies to increase the number of effector sRNAs, also called ‘secondary’ sRNAs, from an initially small pool of ‘primary’ sRNAs that acted as triggers.

In the germline of insects and mammals, a specific class of sRNAs named piRNA are the effectors of RNAi (4,5). piRNAs bind to AGO proteins from the PIWI-clade and are amplified through the ping-pong cycle (6), which occurs at perinuclear germ granules known as Nuage. Recognition of the TE RNA by a primary RISC results in TE RNA target cleavage. The cleavage product is loaded into a different PIWI protein and subsequently trimmed by the conserved 3′–5′ exoribonuclease Nibbler to become a mature secondary piRNA. The new RISC can then recognize and cleave piRNA precursor transcripts, which are then processed by Nibbler to mature piRNAs (7–9).

Nematodes like *Caenorhabditis elegans* employ a different strategy to amplify sRNAs, centred on RNA-dependent RNA polymerases (RdRPs) (10). In the *C. elegans* germline, the RdRP RRF-1 localizes at perinuclear germ granules named Mutator foci, which are thought to be the site of sRNA amplification (11–13). The formation of Mutator foci depends on the intrinsically disordered scaffold protein MUT-16, which acts as a hub for the recruitment of RRF-1 and other proteins to form the Mutator complex (11,13). Currently, more than 12 factors have been associated with the Mutator complex and contribute to sRNA amplification. According to the current model, recognition of the target mRNA by the primary RISC results in target cleavage by the endoribonuclease RDE-8 (14). Subsequently, the nucleotidyltransferase MUT-2 (also called RDE-3) adds an untemplated polyUG (pUG) tail to the 3′ end of the newly generated 5′ fragment, marking it for sRNA amplification (14–16). This pUG tail is thought to act as a signal for the recruitment of the RdRP RRF-1 to the mRNA fragment that is then used as a template to synthesize a pool of secondary 22G sRNA (17–19). These 22G sRNAs are 22 nucleotides (nt) long and carry a characteristic guanosine-triphosphate at the 5′ end (19,20). Mutations in any Mutator component reduce the level of 22Gs and result in RNAi-resistance and transposon activation in the germline (11,19,21–23). While the above model assigns functions to some of the factors of the Mutator complex, the role of most components remains unknown. One of the Mutator factors of yet unknown function is the 3′–5′ exoribonuclease MUT-7, the homologue of *Drosophila melanogaster* Nibbler (22). MUT-7 has been proposed to be recruited to MUT-16 by the nematode-specific factor MUT-8 (also called RDE-2) based on *in vivo* studies (11,24). MUT-8 has no previously annotated domains, and the mechanism by which it recruits MUT-7 to Mutator foci remains unclear.

Although MUT-7 is highly conserved from sponges to mammals (8), a function has only been attributed to *Drosophila* Nibbler, which trims the 3′ end of pre-piRNAs as part of the ping-pong cycle, but also of subpopulations of miRNAs (8,9,25,26). Nibbler is a two-domain protein with an N-terminal domain (NTD) followed by an exoribonuclease (EXO) domain. The NTD consists of a scaffold of HEAT-like repeats with basic patches important for RNA binding and has been proposed to be essential for recruiting Nibbler to AGO-bound RNA substrates (27). The Nibbler EXO domain belongs to the DEDDy family of 3′-to-5′ exoribonucleases (InterPro: IPR002562) and is specific to single-stranded RNA (27).

In addition to these NTD and EXO domains, *C. elegans* MUT-7 carries an additional C-terminal domain (CTD), absent in Nibbler. This domain is classified as MUT7-C (InterPro: IPR002782) and is predicted to be a deteriorated version of the PIN (PilT N-terminal domain)-like domain fold with an inserted zinc finger at the C-terminus (28,29). MUT7-C is evolutionarily ancient, being also found as an individual protein in archaea and bacteria. In some bacteria, like *M. tuberculosis*, the MUT7-C domain is combined with an N-terminal ubiquitin-like domain (Rv0579) (28,29). However, to date, no structure of a MUT7-C has been determined, and no function has been assigned.

Here, we show that most Nibbler/MUT-7 homologues carry a MUT7-C domain and that this domain was lost specifically in drosophilids. We demonstrate that MUT7-C contributes to RNA binding in MUT-7 and its human homologue EXD3. Consequently, deletion of this domain slows down RNA degradation. MUT-7 carries a worm-specific insertion in MUT7-C, which acts as a binding platform for the nematode-specific factor MUT-8. MUT-8, in turn, directly contacts the Mutator scaffold MUT-16, recruiting MUT-7 to Mutator foci. Mutations disrupting the MUT-7/MUT-8 interaction prevent MUT-7 recruitment to Mutator foci and lead to RNAi resistance. These findings assign two functions to the previously uncharacterized MUT7-C domain: an RNA binding function, which most probably represents the most ancient and conserved function and a protein binding platform function that mediates localization.

## Materials and methods

### Cloning and protein production

The genes coding for CeMUT-7 (P34607), CeMUT-8 (Q19672), CeMUT-16 (O62011), HsEXD3 (Q8N9H8) and DrEXD3 (A0A8M9Q6C2) as well as the respective truncations were cloned into modified pET vectors using ligation independent cloning. DrEXD3 and codon-optimized HsEXD3 cDNAs were synthesized by Twist Bioscience. Mutations were generated by the Quick-Change mutagenesis approach. Proteins were produced as fusion proteins with varying fusion tags in the *Escherichia coli* BL21 (DE3) derivatives strain in Terrific Broth (TB) medium. To reconstitute FL and truncated MUT-7/MUT-8 and MUT-8/MUT-16 complexes, MUT-7 was co-expressed with MUT-8 and MUT-8 with MUT-16, respectively. Protein production was induced at 18°C by adding 0.2 mM IPTG for 12–16 h. An overview of the constructs used in this study and the purification steps used is provided in Supplementary Table S1. Proteins were stored at −70°C, usually in 20 mM HEPES/NaOH (pH 7.5), 150 mM NaCl, 2 mM DTT, with or without 10% (v/v) glycerol. 0.5 mM TCEP was used as a reducing agent instead of the DTT for constructs containing the MUT7-C domain with the zinc finger. EXD3 constructs were stored in 20 mM HEPES/NaOH (pH 7.5), 500 mM NaCl, 0.5 mM TCEP.

### Co-expression pulldown assays

For interaction studies by the co-expression co-purification strategy, two plasmids containing the genes of interest and different antibiotic resistance markers were co-transformed into BL21(DE3) derivative strains to allow co-expression (Supplementary Table S2). Cells were grown in 50 ml TB medium shaking at 37°C, and protein production was induced at 18°C by adding 0.2 mM IPTG for 12–16 h. Cell pellets were resuspended in 4 ml of lysis buffer (50 mM NaH_2_PO_4_, 20 mM Tris/HCl, 250 mM NaCl, 10 mM imidazole, 10% (v/v) glycerol, 0.05% (v/v) IGEPAL, 5 mM 2-mercaptoethanol, pH 8.0). Cells were lysed by sonication, and insoluble material was removed by centrifugation at 21 000 × *g* for 10 min at 4°C. 500 μl supernatant was applied to 35 μl amylose resin (New England Biolabs) or glutathione resin (Cytiva) and incubated for 1–2 h at 4°C. Subsequently, the resin was washed three times with 500 μl lysis buffer. The proteins were eluted in 50 μl of lysis buffer supplemented with 10 mM maltose (amylose resin) or 20 mM of reduced glutathione (glutathione resin), respectively. Input and eluate fractions were analysed by SDS-PAGE and Coomassie staining.

### Pulldown assays with purified proteins

To analyse protein interaction with purified proteins, proteins (5 μM) (Supplementary Table S1) were pre-incubated in 50 μl binding buffer containing 20 mM HEPES/NaOH (pH 7.5), 150 mM NaCl, 10% (v/v) glycerol, 0.05% (v/v) IGEPAL, 2 mM DTT for 30 min at 4°C. Samples were added to 20 μl glutathione resin (Cytiva) and gently shaken for 1–2 h at 4°C. Beads were then washed three times with 200 μl binding buffer, and the retained material was eluted with 50 μl incubation buffer supplemented with 20 mM of reduced glutathione. Input material and one-third of the eluate were analysed by SDS-PAGE and Coomassie staining.

### Crystallization

Crystallization trials with the MUT-7^CTD(633–899)^/MUT-8^CTD(322–567)^ complex in SEC buffer (20 mM HEPES/NaOH, pH 8.5, 150 mM NaCl, 0.5 mM TCEP) were performed using a vapour diffusion set-up by mixing the sample and crystallization solution in a 1:1 and 2:1 ratio. Initial needle-shaped crystals were obtained at a protein concentration of 8 mg/ml in condition E3 (0.2 M NaI, 20% (w/v) PEG 3350) from the PACT screen (Molecular Dimensions). We obtained single, plate-shaped crystals through two rounds of cross-matrix micro-seeding using the Morpheus screen (Molecular Dimensions). The best crystals grew in condition A10 (10% (w/v) PEG 8000, 20% (v/v) ethylene glycol, 0.3 M MgCl_2_, 0.3 M CaCl_2_, 0.1 M bicine/Trizma base, pH 8.5). Crystals from this condition did not require further cryoprotection and were, therefore, directly frozen in liquid nitrogen before data collection at 100 K.

### Data processing, phase determination, refinement and model building

Diffraction data were processed automatically by the Grenoble Automatic Data Processing (GrenADES) pipeline from ESRF (https://www.esrf.fr/UsersAndScience/Experiments/MX/How_to_use_our_beamlines/Run_Your_Experiment/automatic-data-processing). Here, data were integrated with XDS (30) and further processed using pointless and aimless (31,32). The phases were determined by molecular replacement using the AlphaFold model of *C. elegans* MUT-7 C-terminal domain AF-P34607-F1 (residues 639–898) (https://alphafold.ebi.ac.uk/entry/P34607). Molecular replacement was performed with Phaser (33) within Phenix (34). Preceding molecular replacement, the model was prepared with Phenix (process predicted model) to translate the pLDDT values to *B* factors and to remove flexible regions. Following molecular replacement, the model was automatically built with ModelCraft (35,36) within CCP4i2 (37), manually completed with COOT (38) and refined with phenix.refine (39) and refmac5 (40). Model quality was assessed using molprobity (41) and PDB-REDO (42). Data collection and refinement statistics are shown in Supplementary Table S3. Molecular graphics of the structures were created using PyMOL 2.5.2.

### Ribonuclease activity assays

RNAs carrying a 5′-6-fluorescein amidite (5′-FAM) label were purchased from Ella Biotech (Fuerstenfeldbruck, Germany): a 28-mer ssRNA (AUUGCAUCUAAAGUUGAUUGAAGAGUUC), a p(GU)_14_ ssRNA (GUGUGUGUGUGUGUGUGUGUGUGUGUGU), a p(GU)_8_ ssRNA (GUGUGUGUGUGUGUGU) and a p(GU)_14_+(A)_10_ ssRNA (GUGUGUGUGUGUGUGUGUGUGUGUGUGUAAA AAAAAAA). Lyophilized RNAs were resuspended in H_2_O at a final concentration of 100 μM and stored at –70°C. To fold the two p(GU) RNAs, RNAs were diluted in folding buffer (50 mM Tris/HCl, pH 7.5, 100 mM KCl) at a final concentration of 20 μM; samples were heated at 90°C for 3 min and then slowly cooled down decreasing the temperature by 1°C/min to 4°C. Control samples were folded in 50 mM Tris/HCl, pH 7.5, 100 mM LiCl. Different concentrations of recombinant proteins were incubated with 1 μM RNA for 30 min at room temperature in a total volume of 10 μl in a buffer containing 20 mM HEPES/NaOH, 50 mM KCl and 2 mM MnCl_2_ unless stated otherwise. For the cation-dependent activity assay, the buffer contained 5 mM of different divalent cations as indicated. For the pUG RNA degradation assay, the buffer contained 50 mM KCl or LiCl as indicated. For the time course experiments comparing *C. elegans* MUT-7 and *Homo sapiens* EXD3 activities, a buffer with 20 mM HEPES/NaOH, NaCl (150 mM for MUT-7, 250 mM for EXD3) and 2 mM MnCl_2_ was used. To stop the reactions, 10 μl Gel Loading Buffer II (Invitrogen) was added, and samples were incubated at 98°C for 5 min. Reaction products were resolved on home-made 15% TBE–urea gel. Gels were scanned with a Typhoon FLA-9500 imager (GE Healthcare).

### Fluorescence polarization (FP) experiments

A 16-mer ssRNA (GUUGAUUGAAGAGUUC) RNA carrying a 5′-6-fluorescein amidite (5′-FAM) label was purchased from Ella Biotech (Fuerstenfeldbruck, Germany). The lyophilized RNA was resuspended in H_2_O at a final concentration of 100 μM and stored at –70°C. Increasing concentrations of proteins were incubated with 50 nM of RNA in a volume of 20 μl at room temperature for 30 min. For the *C. elegans* proteins, a buffer containing 20 mM HEPES/NaOH (pH 7.5), 150 mM NaCl, 10% (v/v) glycerol and 5 mM EDTA was used. For the *H. sapiens* proteins, the buffer contained 20 mM HEPES/NaOH (pH 7.5) and 250 mM NaCl. The fluorescence polarization data were recorded on a Tecan SPARK plate reader with the following settings: excitation 485 nm, bandwidth 20 nm; Emission 535 nm, bandwidth 25 nm. Milli-polarization (mP) values were normalized by subtracting the mP values of the wells containing the fluorophore-labelled RNA only. Data were analysed using the nonlinear regression Hill equation within GraphPad Prism version 10. The mean of three replicates ± SD is shown.

### Circular dichroism (CD)

Far‐UV CD spectra were measured using a Chirascan plus spectrometer (Applied Photophysics) and a 0.5‐mm path‐length cuvette at 20°C. Samples were prepared at 7.8 μM in 5 mM NaH_2_PO_4_, 80 mM NaF at pH 8.0. Eight spectra between 190 and 260 nm (0.5 nm steps) were collected and averaged. After subtraction of the buffer spectrum and conversion of ellipticity (θ) to mean residue ellipticity (MRE), values were plotted using GraphPad Prism version 10.0.2.

### *In vitro* condensate formation assays

Proteins were diluted to a final concentration of 10 μM in 20 mM Tris/HCl (pH 7.5), 150 mM NaCl, 2 mM DTT and 5% (w/v) PEG6000. MUT-7/MUT-8 was mixed with MUT-16^584–724^. 50 μl were immediately loaded onto a 96-well Greiner sensoplate pre-coated with 1% Pluronic F-127. The plate was incubated on the microscope for 100 min to allow droplet formation. Wells were imaged with a Zeiss Axio Observer Z1 in bright field mode, EC Plan-Neofluar 100x/1.3 Oil M27, Orca Flash 4.0 LT+ Camera, VIS-LED at 50% intensity and 20 ms exposure time. Images were analysed with Fiji/ImageJ (43). Scale bars correspond to 50 μm.

### Multiple sequence alignment (MSA)

The following proteins were selected for the sequence alignment of the MUT7-C domains: *Thermococcus eurythermalis* (A0A097QWN3/1–153) Mut7-C RNase domain-containing protein; *Nitrososphaera viennensis* (A0A060HM51/1–171) Mut7-C RNAse domain-containing protein; *Mycobacterium tuberculosis* (O53776/1–252) Twitching motility protein PilT; *Chlamydiota bacterium* (A0A7 × 5Q8E0/1–151) Mut7-C RNAse domain-containing protein; *Arabidopsis thaliana* (A0A1P8BGD5/1–516) 3′–5′ exonuclease domain-containing protein; *Glycine max* (A0A0R0GNL3/1–505) 3′–5′ exonuclease domain-containing protein; *Homo sapiens* (Q8N9H8/1–876) Exonuclease mut-7 homologue; *Gorilla gorilla gorilla* (G3S583/1–764) Exonuclease 3′–5′ domain containing 3; *Canis lupus familiaris* (A0A8P0SNB1/1–905) Exonuclease 3′–5′ domain containing 3; *Danio rerio* (A0A8M9Q6C2/1–861) Exonuclease 3′–5′ domain-containing 3; *Aedes aegypti* (A0A7N4YH63/1–944) Uncharacterized protein; *Musca domestica* (A0A1I8NEK9/1–911) Exonuclease mut-7 homologue; *Caenorhabditis elegans* (P34607/1–910) Exonuclease mut-7. The MSA was performed with ClustalO (44) using standard parameters and was visualized with Jalview (45).

### Maintenance and generation of worm strains

Worm strains were cultured according to standard laboratory conditions at 20°C on nematode growth medium (NGM) plates seeded with *E. coli* OP50 (46). A list of the strains used in this study is provided in Supplementary Table S4.

CRISPR gRNAs were designed using Integrated DNA Technologies CRISPR–Cas9 guide RNA design tool. Strains were obtained by injecting a recombinant Cas9 protein (home-made), a single-stranded primer as a template for homologous recombination (IDT) and a guide RNA molecule (IDT) as previously described (47). The guide AACCTAATTTGGCTTTATTTAGG and the repair template TTCAATTCCATCTGGCAGATTGGTGTGAAGTGCCTCTTGCTCTCTAAATAAAGCCAAATTAGGTTTTAATAATATT were used to generate the *mut-7* (xf367[*mut-7*(R853E, T855E)]) III strain (RFK1714, Supplementary Table S4). The guide AAGCTTGTCCGTTTCGAAAATGG together with the repair template TTTCGTTAGCTTTCTTTTGTACGGTTCTTCTTCACTACCTCCACCTCCACTACCTCCACCTCCACTACCTCCACCTCCGGCATAGTCTGGAAC GTCATATGGGTAAGCGTAATCTGGGACATCG TATGGATACATTTTCGAAACGGACAAGCTTGATTCTGTAAA were used to generate N-terminally HA-tagged MUT-7 strains (RFK1754 and RFK1755, Supplementary Table S4). A (GGGGS)x3 linker was placed between the HA-tag and MUT-7. Mutants were analysed by sequencing and outcrossed two times to wild type N2 worms to remove all potential off-targets.

### RNAi

Adult animals were bleached to obtain embryos, which then hatched and synchronized in M9 buffer (22 mM KH_2_PO_4_, 42 mM Na_2_HPO_4_, 86 mM NaCl, 1 mM MgSO_4_) for 24 h at 20°C. L1 animals were fed with bacteria carrying an empty vector (L4440) or a vector expressing dsRNA against *pos-1* that came from Ahringer library. Young adult worms were transferred to fresh RNAi plates for the egg-laying assay, remaining continuously exposed to RNAi bacteria. All embryos within the first 24 h of egg-laying were scored for hatching. The percentage of viable progeny (number of hatched larvae/total number of laid eggs) was calculated. The progeny of around 15–20 worms was analysed for each strain.

### Western blotting

Synchronized L4 *C. elegans* were harvested and 120 worms were used per sample. Samples were lysed in NuPAGE LDS sample buffer with 100 mM DTT and loaded on a 4–12% Bis–Tris polyacrylamide gel (Thermo Fisher). After transfer onto a PVDF membrane, samples were probed with mouse anti-HA (Sigma–Aldrich, H3663, diluted 1:2000) and rabbit anti-histone H3 (Sigma–Aldrich, H0164, diluted 1:4000) antibodies. HRP-labelled anti-mouse IgG (Cell Signaling, 7076S, diluted 1:3000) and HRP-labelled anti-rabbit IgG (Cell Signaling, 7074S, diluted 1:3000) antibodies were used as secondaries. Membrane was imaged using the Thermo Scientific SuperSignal West Atto kit.

### Immunofluorescence

*C. elegans* at the late L4 stage were dissected in Egg buffer (25 mM HEPES, pH 7.4, 118 mM NaCl, 48 mM KCl, 2 mM EDTA, 0.5 mM EGTA, 10mM NaN_3_) containing 0.1% Tween-20 and fixed in 0.3% formaldehyde in Egg buffer. After freeze-cracking with dry ice, samples were placed in pre-chilled acetone for 20 min. Samples were incubated with primary anti-HA 1:1000 (Sigma–Aldrich, H3663) and secondary anti-mouse IgG Alexa Fluor 555 1:1000 (Thermo Fisher A11029) antibodies. Confocal imaging was performed on a Leica Stellaris 8 falcon confocal microscope using a 40×/1.3 oil-immersion objective. Images were processed with Leica LAS software, ImageJ and Omero.

### AlphaFold predictions

To investigate the RNA-binding mode of MUT-7, EXD3 and *T. eurythermalis* Mut7-C RNase domain-containing protein, we used the AlphaFold server (https://alphafoldserver.com/) (48).

## Results

### MUT-7 interacts with the mutator factor MUT-8

**Figure 1.**
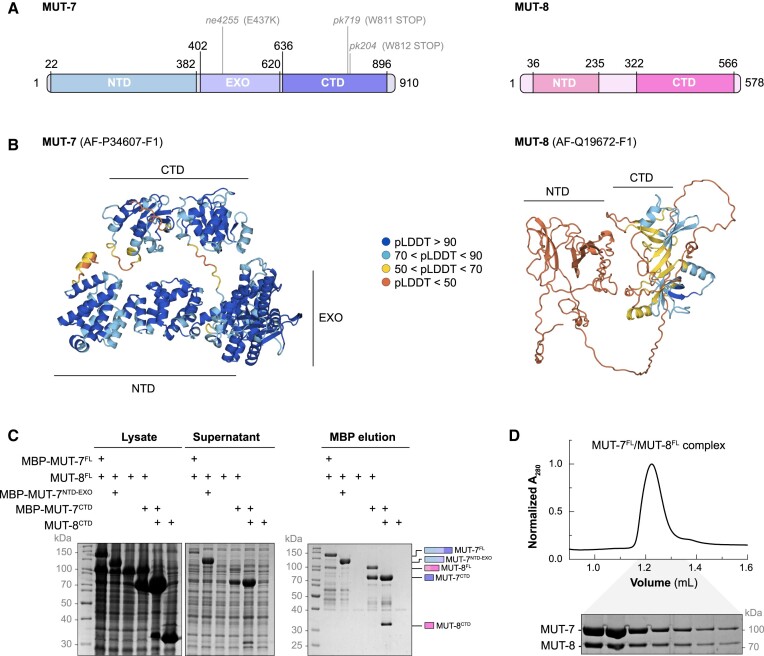
MUT-7 forms a complex with the Mutator factor MUT-8. (**A**) Schematic drawing of MUT-7 (P34607) and MUT-8 (Q19672) proteins. Coloured rectangles highlight domains. Worm mutant strains mentioned in this study are indicated. (**B**) AlphaFold (AF) prediction of MUT-7 (AF-P34607-F1) and MUT-8 (AF-Q19672-F1). Proteins are coloured according to the pLDDT score of their AlphaFold prediction. A high score indicates high confidence of the AF model, while a low score corresponds to low model confidence. (**C**) Analysis of the interaction between MUT-7 and MUT-8 constructs by MBP-pulldown assays. MBP-tagged MUT-7^FL^, MUT-7^NTD-EXO^ and MUT-7^CTD^ were co-expressed with MUT-8^FL^ or MUT-8^CTD^ in *E. coli*. Expression of the preys alone (MUT-8^FL^ and MUT-8^CTD^) was used as a negative control. Total lysate, supernatant and elution are analysed by SDS-PAGE, followed by Coomassie staining. (**D**) Size exclusion chromatography analysis of the MUT-7^FL^/MUT-8^FL^ complex on a Superdex 200 increase 3.2/300 column. The peak fractions are visualized on a Coomassie-stained SDS polyacrylamide gel.

MUT-7 has been reported to interact with the nematode-specific factor MUT-8 by co-immunoprecipitation and yeast two-hybrid experiments (24). To understand the mode of interaction between MUT-7 and MUT-8, we analysed the AlphaFold prediction of the two proteins. The exoribonuclease MUT-7 shows an extended, folded N-terminal domain (NTD) followed by an exoribonuclease (EXO) domain and a C-terminal domain (CTD), which is connected to the EXO domain by a flexible linker (Figure 1A and B). MUT-8 is predicted to be mostly unstructured, except for a partially structured NTD that is connected to a structured CTD by a long, flexible linker (Figure 1A and B). While the folded domains of MUT-7 are all predicted with high confidence, all regions of MUT-8 are predicted with low confidence (Figure 1B), potentially due to its restriction to the *Caenorhabditis* genus (Supplementary Figure S1). Previous yeast two-hybrid experiments showed that MUT-7 residues 643–910 are sufficient to interact with MUT-8 residues 286–578 (24). These minimal constructs roughly correspond to the MUT-7 CTD and MUT-8 CTD. To test if MUT-7 directly binds MUT-8 and if the two CTDs mediate the interaction, we performed co-expression pulldown assays with the following constructs: MUT-7^FL^ (1–910), MUT-7^NTD-EXO^ (1–625), MUT-7^CTD^ (633–910), MUT-8^FL^ (1–578) and MUT-8^CTD^ (322–578). MUT-7 constructs contained an N-terminal MBP-tag and served as bait. MUT-8^FL^ co-precipitated with MUT-7^FL^ but not with MUT-7^NTD-EXO^, and both MUT-8^FL^ and MUT-8^CTD^ also co-precipitated with MUT-7^CTD^ (Figure 1C). This suggests that the two full-length proteins interact directly and that MUT-7^CTD^ and MUT-8^CTD^ mediate the interaction. We note that MUT-8^FL^ and MUT-8^CTD^ are insoluble without MUT-7, suggesting MUT-8′s solubility depends on MUT-7 (Figure 1C). To corroborate those findings, we purified the MUT-7^FL^/MUT-8^FL^ complex through several chromatographic steps, showing that MUT-7^FL^ and MUT-8^FL^ co-migrate in size exclusion chromatography (SEC) (Figure 1D). Analysis of the purified minimal MUT-7^CTD^/MUT-8^CTD^ complex by SEC-Multi-Angle Light Scattering showed a single peak and an average molecular mass of 60 kDa, confirming that MUT-7^CTD^ and MUT-8^CTD^ form a heterodimer (Supplementary Figure S2A).

### Crystal structure of the MUT-7^CTD^/MUT-8^CTD^ complex

Initial attempts to crystallize the MUT-7^CTD^/MUT-8^CTD^ heterodimer were unsuccessful. Limited proteolysis with different proteases resulted in a similar degradation pattern, with MUT-8^CTD^ left intact and MUT-7^CTD^ cleaved into two shorter fragments (Supplementary Figure S2B and C). MUT-8^CTD^ and the two MUT-7^CTD^ fragments co-migrate during SEC, indicating that the complex is stable (Supplementary Figure S2D). Further trimming of a few external amino acids on the CTDs of MUT-7 and MUT-8 yielded diffracting crystals with one copy of the complex in the asymmetric unit. The phases were determined by molecular replacement using the MUT-7^CTD^ AlphaFold model and refined with an *R*-free of 22.1%, *R*-factor of 18.8%, and good stereochemistry (Supplementary Table S3, PDB ID: 8Q66).

**Figure 2.**
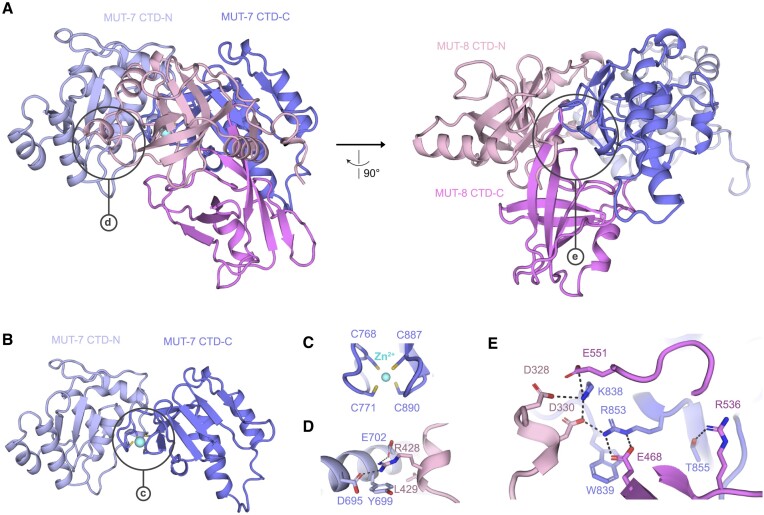
Crystal structure of the MUT-7^CTD^/MUT-8^CTD^ complex. Cartoon representation of the MUT-7^CTD^/MUT-8^CTD^ complex (PDB ID: 8Q66). The respective CTD-N and CTD-C subdomains are shown in shades of blue (MUT-7^CTD^) or pink (MUT-8^CTD^). The Zn^2+^ ion is in cyan. (**A**) Two different orientations of the complex. Black circles highlight the main interaction surfaces between MUT-7^CTD^ and MUT-8^CTD^, of which a close-up view is shown in panels (D) and (E). (**B**) Cartoon visualization of MUT-7^CTD^. A black circle highlights the Cys4 zinc finger, of which a close-up view is shown in panel (C). (**C**), Close-up view of MUT-7^CTD-C^ Cys4 zinc finger. (**D**) Close-up view of the interaction between MUT-7^CTD-N^ and MUT-8^CTD-N^. (**E**) Close-up view of the interaction between MUT-7^CTD-C^ and MUT-8^CTD^.

MUT-7^CTD^ and MUT-8^CTD^ are elongated molecules interacting via an extended interface of 2140 Å^2^ (Figure 2A). The MUT-7^CTD^ can be subdivided into an N-terminal domain (MUT-7^CTD-N^ (633–767)) and a C-terminal domain (MUT-7^CTD-C^ (768–899)) containing a zinc finger with four cysteines coordinating a Zn^2+^ ion (Figure 2B and C). We used Foldseek (49) and DALI (50) to identify domains with structural similarity. MUT-7^CTD-N^ is similar to the response regulator receiver protein from *B. phymatum* (51) and the TOPRIM domain of RNase M5 from *G. stearothermophilus* (52) (Supplementary Figure S3A–C). For MUT-7^CTD-C^, we could not find structures with a Template Modelling (TM)-score higher than 0.4, suggesting that this fold is not yet represented by experimentally determined structures in the PDB (Supplementary Figure S3A). Previous bioinformatic analysis based on sequence comparison had described MUT-7 CTD as a deteriorated version of the PIN-like domain with an additional zinc finger fused at the C-terminus (28). Structural comparison of MUT-7^CTD-N^ against the PDB with Foldseek (49) and DALI (50) did not identify any structurally related PIN domains. Manual comparison of the MUT-7^CTD-N^ with two representative PIN domains from Nob1 and VapC (PDB: 6TG6 and 4CHG) using TM-align (53) resulted in TM-scores of around 0.3, indicating that they are only distantly related.

The MUT-8^CTD^ can also be divided into two subdomains, MUT-8^CTD-N^ and MUT-8^CTD-C^ (Figure 2A and Supplementary Figure S1). Structural analysis using Foldseek (49) revealed that both subdomains show similarity to oligonucleotide/oligosaccharide-binding (OB)-folds flanked by additional secondary structure elements (Supplementary Figure S3A and D–G). OB-fold domains can mediate protein–RNA as well as protein–protein interactions.

Both the MUT-7 and MUT-8 CTD-N and CTD-C subdomains contribute to complex formation. On one side, a short helical insertion in MUT-8^CTD-N^ (Arg428, Leu429) interacts with MUT-7^CTD-N^ (Asp695, Tyr699 and Glu702) (Figure 2A and D). This interface is supported by cross-linking mass spectrometry of the MUT-7^CTD^/MUT-8^CTD^ complex, which shows cross-links between Lys701 of MUT-7 and Thr425 or Lys426 of MUT-8 (Supplementary Figure S3E). On the other side, MUT-7^CTD-C^ forms an extended interaction network with both MUT-8^CTD-N^ and MUT-8^CTD-C^. MUT-7 Arg853 interacts electrostatically with MUT-8 Glu468 and Asp330. MUT-8 Asp330, in turn, interacts with MUT-7 Lys838, which is further stabilized by the interaction with MUT-8 Asp328 and Glu551. Next to MUT-7 Arg853, Thr855 forms a hydrogen bond to MUT-8 Arg536 (Figure 2A and E). Overall, MUT-7^CTD-N^ and MUT-7^CTD-C^ contribute to MUT-8 binding, with MUT-7^CTD-C^ playing a major role. We tested if MUT-7^CTD-C^ was sufficient for MUT-8^CTD^ binding by co-expressing MUT-8^CTD^ with either MBP-tagged MUT-7^CTD^ or MUT-7^CTD-C(773–910)^ and analysed the interaction by pulldown assays. The MUT-8^CTD^ co-precipitated poorly with MUT-7^CTD-C(773–910)^, indicating that MUT-7^CTD-C^ is necessary but insufficient for binding MUT-8 (Supplementary Figure S3H).

Overall, these results give structural insights into a MUT7-C domain and assign a protein-binding function to this previously uncharacterized domain.

### MUT-8 bridges MUT-7 to the Mutator scaffold protein MUT-16

**Figure 3.**
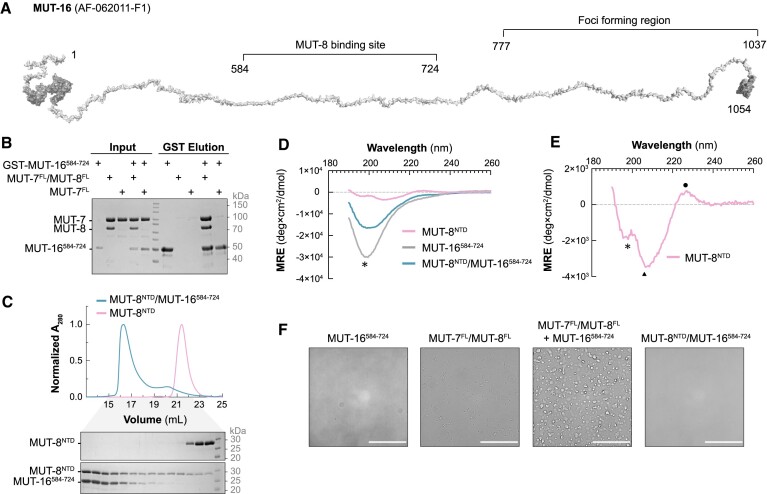
The ternary complex MUT-7/MUT-8/MUT-16 forms condensates. (**A**) MUT-16 AlphaFold prediction shown as cartoon (AF-O62011-F1), stretched with PyMOL. Folded regions are in dark grey and disordered regions in light grey. The MUT-8 binding region identified in this study (584–724) and the foci-forming region (777–1037) are highlighted. (**B**) GST pulldown assay with purified proteins to test the interaction between GST-tagged MUT-16^584–724^ and MUT-7^FL^ or the MUT-7^FL^/MUT-8^FL^ complex. Proteins were incubated with glutathione-coupled beads. Input and elution fractions were analysed by SDS-PAGE followed by Coomassie staining. (**C**) Size exclusion chromatography analysis of purified MUT-8^NTD^ and the complex MUT-8^NTD^/MUT-16^584–724^ on a Superdex 200 increase 10/300 GL column. Chromatograms: MUT-8^NTD^ (pink), MUT-8^NTD^/MUT-16^584–724^ (petrol green). The Coomassie-stained SDS polyacrylamide gels show the peak fractions from size exclusion chromatography. (**D** and **E**) Far-UV CD spectra of MUT-8^NTD^ (pink), MUT-16^584–724^ (grey) and the MUT-8^NTD^/MUT-16^584–724^complex (petrol green). MRE = mean residue ellipticity. A zoom-in on the CD spectrum of MUT-8^NTD^ is shown in panel (E). Peaks with a minimum at 197/198 nm, indicative of an intrinsically disordered protein, are marked by an asterisk. The weak positive peak at 226 nm and a pronounced minimum near 207 nm, indicative of polyproline II conformation, are marked by a circle and a triangle, respectively. (**F**) Phase separation assays using bright-field imaging at a protein concentration of 10 μM in the presence of 5% (w/v) PEG6000 as a crowding agent. The proteins used are indicated above the image. Scale bars correspond to 50 μm.

MUT-7 and MUT-8 co-immunoprecipitate and co-localize with the Mutator scaffold factor MUT-16 and are therefore considered part of the Mutator complex (11,13,54). Based on the observation that MUT-7 fails to localize at germ granules in a *mut-8(pk1657)* mutant strain (11), MUT-8 has been proposed to recruit MUT-7 to Mutator foci. However, no direct interaction between MUT-16 and MUT-8 or any other Mutator component has yet been shown. Of note, two MUT-16 isoforms are annotated: B0379.3a.1 (UniProt: O62011) and B0379.3b.1 (UniProt: Q9U3S5), the latter lacking four amino acids (122-Arg-Asp-Leu-Gln-125). Here, we use the longer B0379.3a.1 isoform as a reference and adapted nomenclature accordingly when referring to experiments using B0379.3b.1 as a reference (11,13). The AlphaFold prediction shows that MUT-16 is mostly disordered with only two structured domains, one at the N-terminus and a small one at the C-terminus (Figure 3A). A region comprising the C-terminal part of the intrinsically disordered region together with the folded C-terminal domain (777–1037) is necessary and sufficient for foci formation in worms (13).

To test if MUT-16 directly contacts the MUT-7/MUT-8 complex and to determine the interacting regions, we performed pulldown experiments. As we failed to express full-length MUT-16, we designed three C-terminally truncated MUT-16 constructs (1–724, 1–584 and 1–383) based on multiple sequence alignment (Supplementary Figure S4) and purified them as GST-tagged fusion proteins for pulldown experiments with full-length MUT-7/MUT-8. While the longest MUT-16 construct (MUT-16^1–724^) co-precipitated MUT-7/MUT-8, the two shorter MUT-16 constructs (MUT-16^1–584^ and MUT-16^1–383^) failed to bind MUT-7/MUT-8 (Supplementary Figure S5A), suggesting that the MUT-16 region 584–724 is essential for MUT7/MUT-8 binding. We purified MUT-16^584–724^ and tested its binding to MUT-7^FL^/MUT-8^FL^ in pulldown experiments. MUT-7^FL^/MUT-8^FL^ co-precipitated with GST-tagged MUT-16^584–724^ as efficiently as with MUT-16^1–724^, indicating that this MUT-16 region is necessary and sufficient for the interaction (Figure 3B and Supplementary Figure S5B). In contrast to full-length MUT-7/MUT-8, the MUT-7^CTD^/MUT-8^CTD^ complex did not bind MUT-16^584–724^, indicating that MUT-16 binding is not mediated by the CTD of either MUT-7 or MUT-8 (Supplementary Figure S5B).

To investigate if MUT-7/MUT-8 interaction with MUT-16 depends on MUT-8, we purified MUT-7^FL^ and tested its binding to GST-MUT-16^584–724^. Without MUT-8, MUT-7^FL^ did not co-precipitate with MUT-16^584–724^ (Figure 3B). Our results indicate that MUT-8 is necessary for MUT-16 binding, but MUT-8 C-terminal domain is insufficient, suggesting that upstream regions might mediate MUT-16 binding. According to AlphaFold, MUT-8 has a partially structured NTD (36–235) (Figure 1B), which we hypothesized interacts with MUT-16. Co-expression of MUT-16^584–724^ with MBP-tagged MUT-8^FL^, MUT-8^NTD^ (1–235) or MUT-8^CTD^ followed by MBP pulldown showed that MUT-16^584–724^ directly binds to the NTD of MUT-8 (Supplementary Figure S5C). Moreover, analysis of the MUT-8^NTD^/MUT-16^584–724^ complex by SEC confirmed the direct interaction of the two constructs (Figure 3C). Quantitative analysis of the interaction between MUT-8^NTD^ and MUT-16^584–724^ using isothermal titration calorimetry revealed a *K*_d_ around 13 μM (Supplementary Figure S5D).

Our findings indicate that MUT-8 uses its folded CTD to bind the MUT-7 CTD and its NTD to contact MUT-16, thus bridging MUT-7 and MUT-16. In agreement with our results, a previous study showed that MUT-8 fails to localize correctly at Mutator foci in worms carrying a MUT-16 deletion in a region (636–776) comparable to the one we identified as MUT-8 binding site (13).

### MUT-7 recruitment to germ granules involves condensate formation

AlphaFold predicts the region of MUT-16 that binds MUT-8 to be fully disordered (Figure 3A), while the MUT-8^NTD^ is predicted to be partially structured, containing some β-strands (Figure 1B).

We purified untagged MUT-16^584–724^ and used circular dichroism (CD) spectroscopy to analyse the secondary structure content of MUT-16^584–724^, MUT-8^NTD^ and the MUT-8^NTD^/MUT-16^584–724^ complex. The far-UV CD spectrum of MUT-16^584–724^ shows a minimum at 197 nm, indicative of an intrinsically disordered region (Figure 3D). The spectrum of MUT-8^NTD^ shows a weak positive peak at 226 nm, a pronounced minimum near 207 nm, and a shoulder at 197 nm (Figure 3D and E). The peaks at 226 and 207 nm are reminiscent of a polyproline II conformation (55), and the shoulder at 197 nm of a disordered polypeptide, suggesting that MUT-8^NTD^ is a mixture of a disordered polypeptide with the presence of regions in a polyproline II conformation. The spectrum of the purified MUT-8^NTD^/MUT-16^584–724^ complex shows a broader minimum at around 200 nm, suggesting that the complex has low secondary structure content. Thus, complex formation seems not to be coupled to secondary structure formation, as the spectrum of the complex can be explained by the sum of the individual spectra (Figure 3D).

Phillips and colleagues showed that the MUT-16 C-terminal region (777–1037) is necessary and sufficient for foci formation *in vivo*. It was also observed that N-terminally extended MUT-16 constructs result in an increased number and size of Mutator foci (13), suggesting that MUT-16 regions upstream of residues 777–1037 might contribute to foci formation. We thus investigated the propensity of MUT-16^584–724^ to form condensates in the presence or absence of MUT-7/MUT-8. While MUT-16^584–724^ and MUT-7^FL^/MUT-8^FL^ on their own did not form condensates, the ternary MUT-7^FL^/MUT-8^FL^/MUT-16^584–724^ complex formed droplets at a concentration of 10 μM in the presence of 5% (w/v) PEG6000 as a crowding agent. Interestingly, the minimal complex MUT-8^NTD^/MUT-16^584–724^ did not form droplets, suggesting additional contributions from the MUT-8^CTD^ or MUT-7 or both to condensate formation (Figure 3F). We conclude that MUT-7/MUT-8 binding to MUT-16 promotes condensation and thereby contributes to Mutator foci formation.

### MUT-7 localization at Mutator foci is required for functional RNA interference

**Figure 4.**
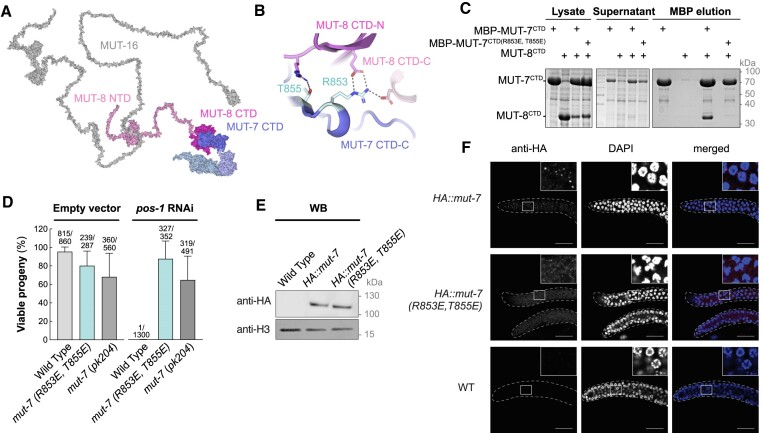
MUT-7 binding to MUT-8 is required for its localization at Mutator foci and functional RNAi. (**A**) Model of MUT-16/MUT-8/MUT-7 complex in cartoon representation. The interaction between proteins has been modelled with PyMOL based on our biochemical results and it is not a prediction. The isolated AlphaFold predictions have been used to create this model. MUT-16 is in grey, MUT-8 in shades of pink, MUT-7 in shades of blue. (**B**) Zoom-in on the MUT-7^CTD^/MUT-8^CTD^ interface, highlighting the mutated residues (cyan) and their interaction network. (**C**) Pulldown assay to test the impact of MUT-7^CTD^ (R853E, T855E) mutations on MUT-8^CTD^ binding. MBP-tagged MUT-7^CTD^ or MUT-7^CTD(R853E, T855E)^ were co-expressed with MUT-8^CTD^ in *E. coli*. Expression of MUT-8^CTD^ alone served as negative control. After lysis, the supernatant is incubated with amylose resin. Total lysate, supernatant and MBP-elution were analysed by SDS-PAGE followed by Coomassie staining. (**D**) Percentage of viable progeny for worms fed with bacteria carrying an empty vector or a vector expressing a dsRNA against *pos-1*. Three *C. elegans* strains were tested: a wild-type (WT) strain, a strain carrying the MUT-7^CTD^ (R853E, T855E) mutation, and the previously characterized *mut-7*(*pk204*) strain. The number of hatched larvae/total number of laid eggs used to calculate the percentage of viable progeny for each strain is indicated above the bars. Error bars represent ± SD of the mean. (**E**) Western blot of whole worm extract from late L4 stage hermaphrodites, using anti-HA or anti-Histone H3 antibodies. Worms expressing HA-MUT-7 (WT or mutant) were analysed. Wild-type worms were used as a negative control. (**F**) Confocal imaging in a mitotic zone of the dissected germline of L4 hermaphrodites, using anti-HA antibodies and DAPI for nuclear staining. Worms expressing HA-MUT-7 (WT or mutant) were analysed. Wild-type worms were used as a negative control. Images are representative of three independent replicates. Scale bars correspond to 20 μm.

Our biochemical data suggest that MUT-8 acts as an adapter, bridging MUT-7 to MUT-16 (Figure 4A) via condensate formation. We reasoned that if MUT-7 and MUT-8 failed to interact, this could prevent the recruitment of MUT-7 to Mutator foci, resulting in the RNAi-resistant phenotype characteristic of Mutator mutants (16,19,21,22,56). To test this, we designed mutations at the MUT-7^CTD^/MUT-8^CTD^ interface that would disrupt MUT-7/MUT-8 interaction (Figure 4B). When MUT-7^CTD^ Arg853 and Thr855 were mutated to glutamate residues, the MUT-8^CTD^ failed to co-precipitate with MBP-tagged MUT-7^CTD^, indicating that the selected mutations disrupt the interaction *in vitro* (Figure 4C). We then introduced these two MUT-7 mutations in worms and tested the resulting strain for RNAi proficiency. As a positive control, we used a worm strain carrying a nonsense mutation in the *mut-7* gene (*pk204*, see Figure 1A), which was previously shown to be RNAi-resistant (21,22). We found that the selected mutations (R853E, T855E) result in fully RNAi-resistant animals (Figure 4D). Mutator foci are still formed in the *mut-7* (R853E, T855E) mutant animals, indicating that the observed phenotype is not due to Mutator foci disruption (Supplementary Figure S5E). To confirm that the observed RNAi-resistant phenotype is due to MUT-7 not being correctly recruited to Mutator foci, we created worm strains expressing HA-MUT-7 (Wild Type or mutant: R853E, T855E). The mutations did not affect the expression level of MUT-7 (Figure 4E). However, mutant MUT-7 (R853E, T855E) no longer localizes to Mutator foci, as demonstrated by immunofluorescence experiments (Figure 4F). These findings indicate that the interaction between MUT-7 and MUT-8 is essential for MUT-7 recruitment to Mutator foci. We conclude that the identified MUT-7 and MUT-8 interface is important for MUT-7 localization and function *in vivo*.

### MUT-7 is a metal-dependent 3′–5′ exoribonuclease

**Figure 5.**
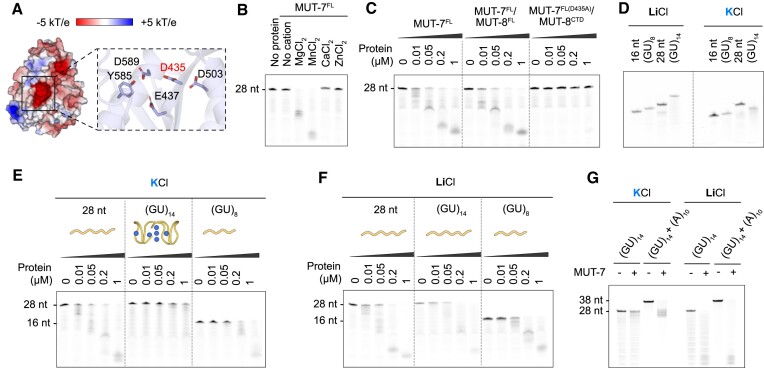
MUT-7 degrades ssRNA but not a pUG fold in a metal-dependent manner. (**A**) Electrostatic surface potential of MUT-7 exoribonuclease domain based on its AlphaFold prediction. Electrostatic surface potential was calculated by Adaptive Poisson–Boltzmann Solver (APBS) from −5 kT/e (red) to +5 kT/e (blue). The zoom-in shows the MUT-7 catalytic site with the mutated Asp435 in inactive MUT-7 highlighted in red. (**B**) Metal ion-dependency of MUT-7 3′–5′ exoribonuclease activity. Purified MUT-7 (0.5 μM) was incubated with a 28-mer ssRNA carrying 5′-FAM-labelled 28-mer ssRNA (1 μM) for 30 min at room temperature in the absence or presence of different divalent cations (5 mM MgCl_2_, MnCl_2_, ZnCl_2_, or CaCl_2_). Reaction products were separated by TBE-Urea PAGE. (**C**) Comparison of MUT-7 and MUT-7/MUT-8 exoribonuclease activity by a dose-dependent ribonuclease assay. An inactive MUT-7^D435A^/MUT-8^CTD^ complex was used as negative control. Increasing amounts of proteins were incubated with a 5′-FAM-labelled 28-mer ssRNA (1 μM) in the presence of 2 mM MnCl_2_ for 30 min at room temperature. Reaction products were separated by TBE-Urea PAGE. (**D**) Native PAGE of indicated 5′-FAM-labelled RNAs in the presence of either 100 mM KCl or LiCl. (GU)_8_ and (GU)_14_ RNAs were pre-folded in folding buffer. A 16-mer and a 28-mer RNAs were used as control. (**E**, **F**) Dose-dependent exoribonuclease assays with various RNA substrates in a buffer containing 50 mM KCl (E) or 50 mM LiCl, preventing pUG fold formation (F). Increasing amounts of MUT-7 were incubated with a (GU)_14_ RNA, a (GU)_8_ RNA that cannot adopt the pUG fold and a 28-mer ssRNA. All RNAs were 5′-FAM-labelled. Reaction products were separated by TBE-Urea PAGE. A cartoon representation of the linear RNAs or the pUG fold (PDB: 7MKT) is shown. K^+^ ions are in blue. (**G**) Exoribonuclease assays with a pre-folded (GU)_14_ RNA carrying a 10 nt A-tail at its 3′-end. A (GU)_14_ RNA was used as control. The assay was performed as described in panels (E) and (F).

Although the role of MUT-7 in the sRNA amplification process remains unknown, its 3′–5′ exoribonuclease activity is necessary for the production of secondary sRNAs, as a mutation in its catalytic site (E437K) leads to TE mobilization (19). The AlphaFold prediction of MUT-7 reveals that the EXO domain adopts an RNase D-like fold with a deep catalytic pocket containing the four conserved residues (DEDD) (Figure 5A), like its *D. melanogaster* homologue Nibbler (27). To test the exoribonuclease activity of MUT-7, we incubated recombinant MUT-7^FL^ with a 5′-6-fluorescein amidite (5′-FAM)-labelled 28-mer ssRNA. Reaction products were detected by fluorescence on 15% TBE-Urea polyacrylamide gels. MUT-7^FL^ showed the highest 3′–5′ exoribonuclease activity in the presence of Mn^2+^, some activity in the presence of Mg^2+^, and no exoribonuclease activity with other divalent metal ions such as Zn^2+^ and Ca^2+^ (Figure 5B), as previously shown for Nibbler (27). We then tested the 3′–5′ exoribonuclease activity of the MUT-7^FL^/MUT-8^FL^ complex to see if MUT-8 impacts MUT-7′s activity. Based on the Nibbler D435A mutant (27), we engineered and purified a catalytically inactive MUT-7^FL(D453A)^/MUT-8^CTD^ complex as a negative control. The MUT-7^FL^/MUT-8^FL^ complex degraded ssRNA with similar efficiency as MUT-7^FL^ alone, while MUT-7^FL(D453A)^/MUT-8^CTD^ was inactive. We conclude that MUT-8 does not affect MUT-7 exoribonuclease activity (Figure 5C).

We next investigated MUT-7 substrate preferences to get hints on MUT-7 putative role in sRNA amplification. In *Drosophila*, Nibbler trims the 3′ end of both miRNAs and piRNAs (8,25,26). Similarly, *C. elegans* MUT-7 might be necessary for trimming RdRP products to their final length of 22 nt. Alternatively, MUT-7 could act on different RNA substrates. A prominent RNA structure that plays a critical role in the *C. elegans* sRNA amplification process is the pUG fold (15,17,18,57). Following template RNA cleavage, the newly generated 3′ hydroxyl is modified by the nucleotidyltransferase MUT-2, which appends repeating dinucleotide UG-units (15). 11.5 UG/GU repeats can form a compact quadruplex (G4) structure named pUG fold in the presence of potassium (K^+^) but not lithium ions (Li^+^) (18,58). To investigate if MUT-7 is able to degrade such a pUG fold, we performed ribonuclease assays with (GU)_8_ and (GU)_14_ RNAs, with the (GU)_8_ RNA not being long enough to adopt a pUG fold (18,58). We first checked the folding of (GU)_8_ and (GU)_14_ RNAs by native PAGE with K^+^ versus Li^+^. As expected, (GU)_14_ migrated faster in the presence of K^+^ compared to Li^+^, while (GU)_8_ did not, indicating that only the (GU)_14_ RNA is able to adopt a compact K^+^-dependent pUG fold (Figure 5D). MUT-7^FL^ efficiently degraded the (GU)_8_ but failed to efficiently degrade the (GU)_14_ RNA in the presence of K^+^ (Figure 5E). However, both (GU)_8_ and (GU)_14_ were degraded in the presence of Li^+^, indicating that the folding of the (GU)_14_ RNA prevents its degradation by MUT-7 (Figure 5F). To further support this, we used a (GU)_14_ RNA substrate with an additional 10 nt A-tail (A_10_) at its 3′-end. As expected, MUT-7 efficiently trimmed the A-tail but failed to degrade the folded pUG unit (K^+^). However, in the presence of Li^+^, both the A-tail and the pUG unit were degraded (Figure 5G). This result would support the intriguing hypothesis that MUT-7 degrades unstructured UG repeats downstream of the folded pUG unit. However, further studies are required to dissect the function of MUT-7 in sRNA amplification.

### The MUT7-C domain is evolutionarily conserved throughout all domains of life

**Figure 6.**
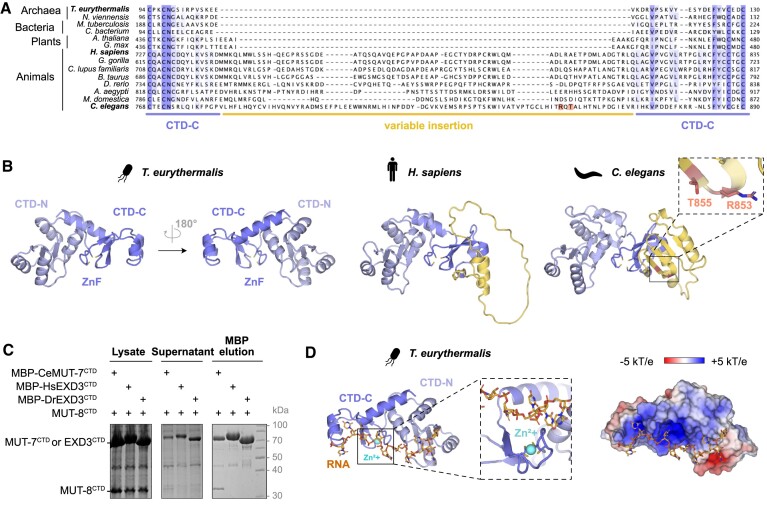
MUT7-C is an evolutionarily ancient domain. (**A**) Multiple sequence alignment of prokaryotic and eukaryotic MUT7-C domains, limited to the sequence between the first and second pair of cysteines of the zinc finger. Residues are marked with shades of blue based on their conservation score. The variable insertions are highlighted with a yellow line, the CTD-C with a blue line. MUT-7^CTD^ residues preventing MUT-8 binding (R853, T855) are in orange. (**B**) Cartoon representation of the MUT7-C domains from three different proteins: *T. eurythermalis* Mut7-C RNase domain-containing protein (AF-A0A097QWN3-F1), *H. sapiens* EXD3 (AF-Q8N9H8-F1) and *C. elegans* MUT-7 (PDB: 8Q66). Two side views are shown for *T. eurythermalis* Mut7-C RNase domain-containing protein. The MUT7-C domains consist of an N-terminal (CTD-N) and a C-terminal part (CTD-C). The CTD-N is shown in light blue, the CTD-C in blue and the insertions in yellow. The four cysteine residues of the zinc finger are shown as sticks. A zoom-in shows *C. elegans* MUT-7 mutated residues (R853, T855) in orange. (**C**) Co-expression pulldown assays testing the species-specificity of the MUT-7^CTD^/MUT-8^CTD^ interaction. MBP-tagged CeMUT-7^CTD^, HsEXD3^CTD^ and DrEXD3^CTD^ were co-expressed in *E. coli* with CeMUT-8^CTD^. Hs: *Homo sapiens*; Dr: *Danio rerio*; Ce: *Caenorhabditis elegans*. After lysis, the supernatant was incubated with amylose resin. Total lysate, supernatant and elutions were analysed by SDS-PAGE followed by Coomassie staining. (**D**) AlphaFold3 prediction of *T. eurythermalis* Mut7-C RNase domain-containing protein with a zinc ion and a 10 nt U10 RNA. The cartoon representation (left) and the electrostatic surface potential (right) of the protein are shown. A zoom-in shows the zinc ion (cyan) and the RNA (orange). The electrostatic surface potential was calculated by Adaptive Poisson–Boltzmann Solver from −5 kT/e (red) to +5 kT/e (blue).

We showed that *C. elegans* MUT-7^CTD^ binds MUT-8^CTD^, which in turn interacts with MUT-16 through its NTD. Hence, one role of the MUT-7 CTD in *C. elegans* is to establish the MUT-7 localization to Mutator foci via MUT-8. Given that both MUT-8 and MUT-16 are conserved in *Caenorhabditis* (Supplementary Figures S1 and S4), this MUT-7 CTD function is likely to be extended to other *Caenorhabditis* species. Interestingly, the only MUT-7 homologue that has been characterized is *D. melanogaster* Nibbler, which lacks the domain classified as MUT7-C. A comprehensive analysis of the presence/absence of the MUT7-C domain in MUT-7 animal homologues has not yet been done. We performed a thorough phylogenetic analysis of the distribution of MUT-7 proteins in animals. This revealed that most animal homologues, including the vertebrate factor EXD3, carry a MUT7-C domain downstream of the EXO domain. The MUT7-C domain is also present in the common ancestors of Nibbler family members, indicating that a loss has occurred in drosophilids (Supplementary Figure S6). Interestingly, in the yellow fever mosquito (*A. aegypti*) Nibbler does carry the MUT7-C (A0A7N4YH63). However, recent work did not consider this domain (27), likely because the previously annotated isoform lacked the CTD (Q179T2). Since Mutator complexes are not present in animals besides *Caenorhabditis* (11,13), this implies that the MUT7-C domain likely has a function beyond mediating MUT-8 binding. So far, no function has been assigned to MUT7-C domains. To identify the core elements of the MUT7-C fold and gain insights into the potentially conserved function of the MUT7-C domain, we generated a multiple sequence alignment including bacterial, archaeal, and eukaryotic sequences (Figure 6A and Supplementary Figure S7A). In parallel, we used the AlphaFold structure of the archaeon *T. eurythermalis* Mut7-C RNase domain-containing protein (A0A097QWN3) as representative of the most minimal MUT7-C fold to visualize the conservation of the different elements (Figure 6B and Supplementary Figure S7B). We compared this structure with the experimental structure of *C. elegans* MUT-7 CTD and the AlphaFold prediction of the CTD from its human homologue EXD3 (Figure 6B). Our analysis revealed that prokaryotes and plants carry a minimal conserved MUT7-C unit consisting of an N-terminal part (CTD-N) and a C-terminal part (CTD-C). A detailed analysis of the core structural elements is shown in Supplementary Figure S7B.

While the CTD-N is evolutionarily highly conserved at both the sequence and structural level (Supplementary Figure S7A and B), the CTD-C exhibits significant variability among species. Animals contain various insertions between the first and second pair of cysteines of the zinc finger (Figure 6A and B). In humans, the insertion is mainly disordered, but in *C. elegans* (and other *Caenorhabditis* species), it forms a four-stranded β-sheet (aa 784–868) (Figure 6B). The surface of the β-sheet participates in MUT-8 binding and mutations in this region (R853E, T855E) abolish complex formation (Figures 4 and 6B). Given the sequence and structural differences of nematode and vertebrate insertions, we hypothesized that the CTDs from *H. sapiens* (Hs) or *D. rerio* (Dr) EXD3 would not bind MUT-8. To test this, we co-expressed the MUT-8^CTD^ with MBP-tagged HsEXD3^CTD^ and DrEXD3^CTD^ and analysed the interaction by pulldown experiments. MUT-8^CTD^ was not soluble when co-expressed with HsEXD3^CTD^ or DrEXD3^CTD^, suggesting that the MUT-8^CTD^ does not interact with the vertebrate orthologue EXD3 and supporting the species-specificity of the MUT-7/MUT-8 interaction (Figure 6C).

Our findings reveal that the evolutionarily conserved MUT7-C domain has acquired insertions that differ significantly across species. While the role of the insertion in other organisms remains unknown, the well-structured insertion present in *Caenorhabditis* mediates binding to MUT-8, thereby allowing recruitment of MUT-7 to Mutator foci.

### The MUT7-C domain has a conserved role in RNA binding

What is the conserved, ancestral function of the MUT7-C domain? The MUT7-C domain contains a zinc finger, a motif often involved in nucleic acid binding. We turned to the newly released AlphaFold server (48), which employs AlphaFold3, to predict the interaction of *T. eurythermalis* Mut7-C RNase domain-containing protein with a zinc ion and a 10 nt RNA. AlphaFold3 predicted with high confidence the interaction between the MUT7-C domain and both zinc (ipTM = 0.97, pTM = 0.9) and RNA (ipTM = 0.65, pTM = 0.88) (Figure 6D). Mapping the electrostatic surface potential of the protein revealed that the RNA is predicted to bind a highly positively charged surface (Figure 6D). Interestingly, the zinc finger does not seem to be directly involved in RNA binding but rather in coordinating the CTD-N and the CTD-C parts of the MUT7-C domain (Figure 6D and Supplementary Figure S7B).

**Figure 7.**
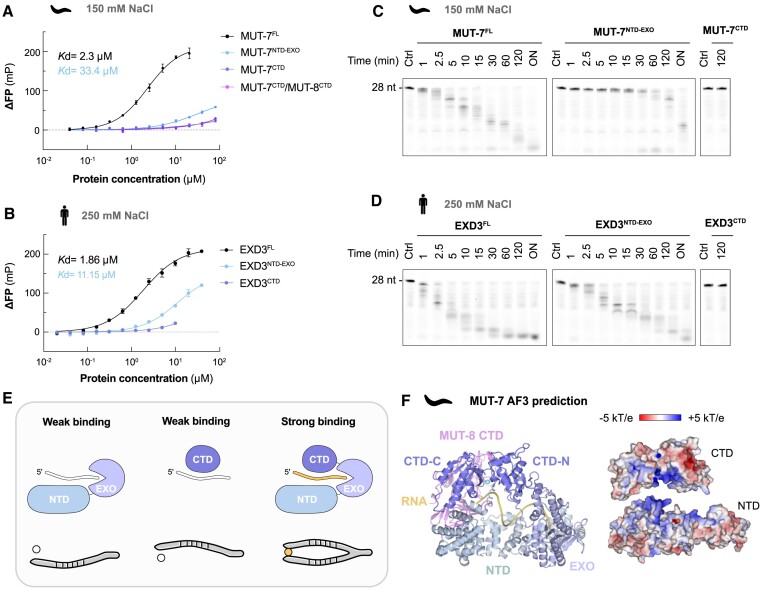
The MUT7-C contributes to the RNA binding properties of MUT-7 and EXD3. (**A**, **B**), RNA-binding affinities of different MUT-7 and EXD3 constructs determined by fluorescence polarization (FP) assays. A 5′-FAM-labelled 16-mer ssRNA (50 nM) was incubated with increasing concentrations of the indicated proteins. FP values were normalized by subtracting the FP values of the wells containing the labelled RNA alone. The mean of three experiments is shown, and error bars correspond to ± SD. The FP data are fitted to the Hill equation to obtain the dissociation constant, *K*_d_. RNA binding of MUT-7^FL^, MUT-7^NTD-EXO^, MUT-7^CTD^ and MUT-7^CTD^/MUT-8^CTD^ was tested in a buffer with 150 mM NaCl and 5 mM EDTA (A). For EXD3^FL(D399A)^, EXD3^NTD-EXO(D399A)^, EXD3^CTD^ and MUT-7^FL(D435A)^/MUT-8^CTD^, a buffer containing 250 mM NaCl was used (B). (**C**, **D**), Time-course assessing the ribonuclease activity of indicated MUT-7 (C) and EXD3 (D) constructs. Proteins (0.5 μM) were incubated with a 5′-FAM 28-mer ssRNA (1 μM) in a buffer containing the indicated NaCl concentration at room temperature for the indicated times. MUT-7^FL^, MUT-7^NTD-EXO^, MUT-7^CTD^, EXD3^FL^, EXD3^NTD-EXO^ and EXD3^CTD^ were tested. Reaction products were separated by TBE-Urea PAGE. (**E**), Model of RNA-binding mode of the exoribonuclease MUT-7. The different domains are in shades of blue. (**F**) AlphaFold3 prediction of MUT-7 bound to MUT-8 CTD, a ssRNA, Mg^2+^ and Zn^2+^. pTM = 0.69, ipTM = 0.89. The MUT-7 domains are in shades of blue, MUT-8 CTD is in pink, RNA in yellow, zinc in cyan, magnesium in green. The electrostatic surface potential was calculated by Adaptive Poisson–Boltzmann Solver from −5 kT/e (red) to +5 kT/e (blue).

These findings prompted us to investigate the contribution of the MUT7-C domain to RNA binding for both worm MUT-7 and human EXD3 by fluorescence polarization (FP) assays using a 5′-FAM-labelled 16-mer ssRNA. For that, we purified the following constructs: MUT-7^FL^, MUT-7^NTD-EXO^, MUT-7^CTD^, catalytically inactive EXD3^FL(D399A)^ (1–876), EXD3^NTD-EXO(D399A)^ (1–582) and EXD3^CTD^ (624–876). We also verified the presence of Zn^2+^ in EXD3^CTD^ by SEC combined with inductively coupled plasma mass spectrometry (Supplementary Figure S8A and B). MUT-7^FL^bound ssRNA with an affinity in the micromolar range (*K*_d_ = 2.3 μM). MUT-7^NTD-EXO^, which lacks the CTD, had a roughly 8-fold weaker affinity (*K*_d_ = 18.7 μM), while the isolated MUT-7^CTD^ only bound RNA weakly. The MUT-7^CTD^/MUT-8^CTD^ complex bound RNA as weakly as the MUT-7^CTD^ (Figure 7A and Supplementary Figure S8C). This suggests that MUT-8^CTD^ does not play a role in RNA binding, which is also supported by the observation that the MUT-7^FL^/MUT-8^FL^ complex degraded RNA with similar efficiency as MUT-7^FL^ alone (Figure 5C). To avoid precipitation of human EXD-3, FP experiments were performed at 250 mM NaCl, while experiments with *C. elegans* MUT-7 were done in a buffer containing 150 mM NaCl. The results for EXD3 were similar to those obtained for MUT-7. The affinity of EXD3^FL(D399A)^ for the ssRNA was in the micromolar range (*K*_d_ = 1.86 μM). EXD3^NTD-EXO(D399A)^, which lacks the CTD, had a roughly 6-fold weaker affinity (*K*_d_ = 11.15 μM), while the EXD3^CTD^ alone only bound RNA weakly (Figure 7B and Supplementary Figure S8D).

To investigate the functional contribution of the CTD to MUT-7/EXD3 ribonuclease activity, we compared the RNA degradation kinetics of MUT-7 and EXD3 in the presence and absence of the CTD. To this end, we performed a time course with a 5′-FAM-labelled 28-mer ssRNA using the following constructs: MUT-7^FL^, MUT-7^NTD-EXO^, and the newly purified active EXD3^FL^ and EXD3^NTD-EXO^. MUT-7^CTD^ and EXD3^CTD^ were used as negative controls. For both MUT-7 and EXD3, the full-length constructs degraded RNA more efficiently than the ones lacking the CTD (Figure 7C and D). Overall, our results suggest that for both worm MUT-7 and human EXD3, the MUT7-C domain synergistically contributes with the NTD and EXO domains to RNA binding. We propose a model where the NTD and the CTD act as two arms of a forceps, which individually cannot efficiently bind RNA (Figure 7E). By contributing to RNA binding, the MUT7-C enhances the RNA degradation kinetics. To support our model, we used AlphaFold3 to predict the binding to RNA for MUT-7 and EXD-3. For MUT-7, we also included MUT-8 CTD. The predictions support our model, showing that both the NTD-EXO and the CTD have a positively charged surface that binds RNA (Figure 7F and Supplementary Figure S8E). The MUT-8 CTD binds to the opposite side of the RNA-binding surface of the MUT-7 CTD (Figure 7F, also see Figure 6D), supporting the biochemical observation that the MUT-8 CTD does not contribute to RNA binding.

## Discussion

In this study, we have structurally and functionally characterized the MUT7-C, an ancient domain belonging to the conserved 3′–5′ exoribonuclease MUT-7 in animals, while in prokaryotes it exists mostly as an individual protein.

Prokaryotes have a reduced, minimal version of the MUT-7C domain consisting of a TOPRIM-like fold and a zinc finger at the C-terminus. We did not detect significant structural similarity with PIN-like domains, as previously suggested, indicating that MUT7-C and PIN domains are only distantly related. Animals contain variable insertions within the zinc finger of the MUT7-C domain. Thus, the MUT7-C appears structurally plastic, suggesting a possible functional adaptation of this domain during evolution.

In *Caenorhabditis*, the MUT7-C insertion differs significantly from other animals at the sequence and structural level. Here, it forms a structured β-sheet, constituting the main binding site for the nematode-specific factor MUT-8. MUT-8 is a modular protein that binds MUT-7 and the Mutator scaffold MUT-16, acting as a bridge and recruiting MUT-7 to Mutator foci. The interaction between MUT-8 and MUT-7 is mediated by the two folded CTDs forming a tight complex. In contrast, the interaction between MUT-8 and MUT-16 is mediated by disordered regions of both proteins: the MUT-8 NTD and an internal MUT-16 segment. Investigation of the MUT-8/MUT-16 interaction by molecular dynamics simulations approaches revealed the importance of cation-pi interactions between Tyr residues from MUT-8 and Arg/Lys residues from MUT-16 (59).

Mutations in the MUT7-C β-sheet prevent MUT-8 binding and result in RNAi resistance. Similarly, worm strains carrying nonsense mutations in MUT-7 CTD upstream of the insertion (*pk719*: W811/* and *pk204*: W812/*, see Figure 1A) show transposon de-silencing and RNAi resistance (19). These mutations result in truncations of the MUT-7 CTD, leading to loss of MUT-8 binding. This strongly suggests that correct localization of MUT-7 at Mutator foci depends on MUT-8 binding and is necessary for sRNA amplification. Consistently, the MUT-7 paralogue ZK1098.3 (P34603), which contains the NTD and EXO domains but only a partial CTD lacking the MUT-8 binding site, does not compensate for MUT-7 mutations.

But what is the role of MUT-7 in the sRNA amplification process at Mutator foci? The 3′–5′ exoribonuclease activity of MUT-7 is necessary for sRNA amplification, as *mut-7* mutants show a drop in 22G RNAs (19,60), and mutations close to (G497E) or in the catalytic site (E437K) lead to RNAi resistance and TE mobilization (19,24). We have shown that *in vitro* MUT-7 only efficiently degrades unstructured RNAs, but not the pUG fold. We could envisage the following roles of MUT-7 in small RNA amplification: (i) after cleavage of the template RNA by RDE-8 (14), MUT-7 could be required for the priming of the newly formed 3′ end, preparing for the addition of the pUG tail by MUT-2. The pUG tail seems to serve two functions at a time: stabilizing the newly formed 3′ end of the 5′ fragment and marking it as a template for the RdRP to initiate sRNA amplification (17). (ii) MUT-7 could remove additional overhanging nucleotides from the pUG fold. (iii) MUT-7 could trim the 3′ of RdRP products to their final length of 22 nt, similar to the function of Nibbler in *Drosophila* (7–9). 4) MUT-7 could degrade abortive RdRP transcripts that are too short to function efficiently in RNAi. However, further studies are necessary to unravel the molecular function of MUT-7.

In the attempt to dissect the ancient function of the MUT7-C domain unrelated to the insertion, we showed that the CTD contributes to RNA binding for both *C. elegans* MUT-7 and the human homologue EXD3. Deletion of the CTD decreases the efficiency of RNA binding and RNA degradation, suggesting that it is functionally important. This might indicate that RNA binding is an evolutionary feature of the MUT7-C in animals and can likely be extended to other MUT-7 homologues. While the ancient function of the MUT-7C domain in archaea and bacteria remains unknown, it is conceivable to speculate that it functions as an RNA binding module. This is supported by the observation that heterologous expression of *M. tuberculosis* MUT-7C homologue Rv0579 in *E. coli* leads to co-purification of nucleic acids (61). Dissecting the function of Rv0579 would be clinically relevant, as a mutation in Rv0579 (L240V) results in resistance to the prodrug TP053 by a yet unidentified mechanism (61).

## Supplementary Material

gkae610_Supplemental_File

## Data Availability

Coordinates and structure factors of the MUT-7^CTD^/MUT-8^CTD^ complex structure have been deposited in the Protein Data Bank (PDB) with accession code PDB ID 8Q66 (https://doi.org/10.2210/pdb8Q66/pdb). The cross-linking mass spectrometry data have been deposited at the PRIDE database under the Project accession PXD048205.
