# Supplementary material for: MUT-7 exoribonuclease activity and localization are mediated by an ancient domain
Source: Nucleic Acids Res. 2024 Jul 23;52(15):9076–91. doi: 10.1093/nar/gkae610 (PMC11347159; doi:10.1093/nar/gkae610)
Supplement: gkae610_Supplemental_File [file gkae610_supplemental_file.pdf]

Supplementary information

MUT-7 exoribonuclease activity and localisation are mediated by an ancient domain

Virginia Busetto[1,2], Lizaveta Pshanichnaya[3,4], Raffael Lichtenberger[1,2], Stephan Hann[5], René F. Ketting[3,6], Sebastian Falk[1,2#].

[1] Max Perutz Labs, Vienna Biocenter Campus (VBC), Dr.-Bohr-Gasse 9, 1030, Vienna, Austria.

[2] University of Vienna, Max Perutz Labs, Department of Structural and Computational Biology, Center for Molecular Biology, University of Vienna, Campus Vienna Biocenter 5, 1030, Vienna, Austria.

[3] Biology of Non-coding RNA group, Institute of Molecular Biology, Ackermannweg 4, 55128 Mainz, Germany.

[4] International PhD Programme on Gene Regulation, Epigenetics & Genome Stability, Mainz, Germany.

[5] University of Natural Resources and Life Sciences, Vienna, Department of Chemistry, Institute of Analytical Chemistry, Muthgasse 18, 1190 Vienna, Austria.

[6] Institute of Developmental Biology and Neurobiology, Johannes Gutenberg University, 55099 Mainz, Germany.

[#] Correspondence: sebastian.falk@univie.ac.at

Supplementary Material and Methods

Crosslinking-Mass Spectrometry

1 mg DSBU (Disuccinimidyl Dibutyric Urea) (ThermoScientific) was resuspended in DMSO to reach a final concentration of 50 mM and stock aliquots were stored at -20 °C. Increasing quantities of DSBU were added to the MUT-7^{CTD}/MUT-8^{CTD} complex (9 μM) in 20 mM HEPES/NaOH pH 7.5, 150 mM NaCl, 2 mM DTT. Reactions were incubated for 30 minutes at room temperature and then stopped by adding 25 mM Tris/HCl (pH 7.5) for 15 min at room temperature. Samples were analysed by SDS-PAGE and the crosslinked band of interest was excised from the gel. The Coomassie-stained gel band was destained with a mixture of acetonitrile and 50 mM ammonium bicarbonate. The proteins were reduced using 10 mM DTT and alkylated with 50 mM iodoacetamide. Trypsin (Mass Spectrometry Grade) was used for proteolytic cleavage. Digestion was carried out with trypsin at 37°C overnight. Formic acid was used to stop the digestion and extracted peptides were desalted using C18 Stagetips (62).

Peptides were separated on an ultimate 3000 nano-flow chromatography system (Thermo-Fisher), using a pre-column for sample loading (Acclaim PepMap C18, 2 cm × 0.1 mm, 5 μm, Thermo-Fisher), and a C18 analytical column (Acclaim PepMap C18, 50 cm × 0.75 mm, 2 μm, Thermo-Fisher), applying a segmented linear gradient from 2% to 35% and finally 80% solvent B with a flow rate of 230 nL/min over 120 minutes. Solvent A: 0.1 % formic acid; Solvent B: 0.1 % formic acid, 80 % acetonitrile.

Eluting peptides were analysed on an Exploris 480 Orbitrap mass spectrometer (Thermo Fisher) using coated emitter tips (PepSep, MSWil) with the following settings: The mass spectrometer was operated in DDA mode with the FAIMS CV set to -40, -55 and -70. The cycle time was set to 1 s. The survey scans were obtained in a mass range of 375-1600 m/z, at a resolution of 120k at 200 m/z, and a normalized AGC target at 100%. The selected ions were isolated with a width of 1.2 m/z, fragmented in the HCD cell at 27%, 30%, 33% collision energy, and the spectra recorded for max. 200 ms at a normalized AGC target of 200% and a resolution of 30k. Peptides with a charge of +3 to +8 were included for fragmentation, the peptide match feature was set to preferred, the exclude isotope feature was enabled, and selected precursors were dynamically excluded from repeated sampling for 30 seconds.

Raw data were processed using the MaxQuant software package 2.0.3.0 (63) and searched against the target sequence, the Uniprot *E. coli* reference proteome (version 2021_03, www.uniprot.org) as well as a database of most common contaminants. The search was performed with standard identification settings: full trypsin specificity allowing a maximum of two missed cleavages. Carbamidomethylation of cysteine residues was set as fixed, oxidation of methionine and acetylation of protein N-termini as variable modifications. All other settings were left at default. Results were filtered at a false discovery rate of 1% at protein and peptide spectrum match level.

To identify cross-linked peptides, the spectra were searched using Merox software 2.0 (64) against the sequences of the top 10 non-contaminant proteins from the MQ search sorted by iBAQ. Carbamidomethylation of cysteine was set as fixed, oxidation of methionine and acetylation of protein N-termini as variable modifications. The enzyme specificity was set to trypsin allowing 4 missed cleavage sites. Crosslinker settings were selected as DSBU. Search results were filtered for 1% FDR (false discovery rate) on the PSM level (peptide-spectrum matches) and a maximum precursor mass deviation of 5 ppm. To remove low quality PSMs, additionally an score cutoff of 50 was applied. Cross-link maps were generated in xiNET (65).

Limited proteolysis

The MUT-7^{CTD}/MUT-8^{CTD} complex at 0.7 mg/mL was incubated with increasing concentrations of Trypsin, Elastase, Chymotrypsin and Glu C (0.02, 0.002 and 0.0002 mg/mL) in 13 µL buffer (20 mM HEPES/NaOH, 50 mM NaCl, 2 mM $MgCl_2$). Reactions were incubated for 30 minutes on ice. A time course was performed with Elastase at 0.02 mg/mL. Reaction products were visualized by SDS-PAGE. The Elastase reaction products were analysed by Size Exclusion Chromatography.

Size-exclusion chromatography coupled to multi-angle light scattering (SEC-MALS)

The molecular mass and the oligomeric state of the MUT-7^{CTD}/MUT-8^{CTD} complex were determined by size exclusion chromatography (SEC) coupled to multi-angle light scattering (MALS). The complex was analysed at a concentration of 5.4 mg/mL in a buffer consisting of 20 mM Tris/HCl pH 7.5, 150 mM NaCl, and 2 mM DTT. A Superdex

75 Increase 10/300 column (GE Healthcare Life Sciences) was connected to a 1260 Infinity HPLC system (Agilent Technologies) coupled to a MiniDawn Treos detector (Wyatt Technologies) with a laser emitting at 690 nm. An RI-101 detector (Shodex) was used for refractive index measurement. Data were analysed using the Astra 7 software package (Wyatt Technologies).

Isothermal titration calorimetry (ITC)

ITC was carried out by using a MicroCal PEAQ-ITC calorimeter (Malvern Panalytical). All samples were dialyzed against a buffer containing 25 mM Tris/HCl pH 7.5, 150 mM NaCl, 0.25 mM TCEP. The cell was filled with 46 µM MUT-8^{NTD}, the syringe with 600 µM MUT-$16^{584-724}$. Titrations were carried out at 20 °C with 19 injections of 2 µL. As control, the injectant was titrated into buffer. All data were processed and curves fitted using the MicroCal PEAQ ITC software.

Microscopy

For live imaging, 20–25 young adult worms were picked to a drop (80 µl) of M9 buffer (22 mM KH_2PO_4, 42 mM Na_2HPO_4, 86 mM NaCl, 1 mM $MgSO_4$) on a slide and washed in M9 with 0.05M NaN_3 to paralyse the worms. After removing M9, a slide prepared with 2% agarose (in water) was placed on top of the coverslip and worms were imaged directly. Images were acquired at a Leica TCS SP5 STED CW confocal microscope (objective HC PL APO CS2 40x 1.3 oil-immersion objective, Leica). Images were processed with Leica LAS software and ImageJ.

Phylogenetic analysis

Orthodb v10.1 and EggNOG v5 were used to derive predicted Nibbler/Mut7 orthologs at various taxonomic levels, Interproscan v5.42-78.0 for the identification of Pfam domains within these sequences, and iTol v6 for visualization of the phylogenetic distribution of the orthologous groups on the phylogenetic species tree (PMID: 33885785).

Size-Exclusion Chromatography-Inductively Coupled Plasma Mass Spectrometry (SEC-ICP-MS)

HsEXD3^{CTD} was purified as described in **Table S1** and stored at -70 °C in 20 mM Tris/HCl (pH7.5), 150 mM NaCl, 10% (v/v) glycerol, 0.5 mM TCEP at a concentration of 1.5 mg/mL prior to measurement. A NexSAR Speciation Analysis Ready HPLC system from Perkin Elmer (Massachusetts, USA) controlled by Clarity 8.8 software was used for the size exclusion separation. The mobile phase consisted of 75 mmol/L NaCl and 10 mmol/L Tris/HCl at pH 7.2 and the flow rate was set to 0.3 mL min^{-1}. The injection volume for injection of samples, blank solutions and standards onto the ACQUITY UPLC Protein BEH 200Å SEC column (Waters) was 15 µL. For elemental detection, the system was combined with a NexION 2000 quadrupole ICP-MS from Perkin Elmer (Massachusetts, USA). The reaction cell was filled with oxygen to induce the formation of $^{32}S^{16}O^+$ and circumvent the $^{16}O_2^+$ interference on the most abundant sulfur isotope ^{32}S. Zinc was detected as ^{66}Zn. Bovine Cu/Zn-superoxide dismutase (SOD) was used to determine the intensity ratio between the ^{66}Zn and the $^{32}S^{16}O$ signals. The stoichiometric zinc ratio was calculated via the number of sulfur atoms per protein. Details concerning the optimization of the ICP-MS parameters and data evaluation can be found in a previous publication (66). pH measurements were conducted with an Education Line EL20 pH-Meter with a micro electrode (inLab Micro, Mettler, Toledo, USA).

Supplementary Figures

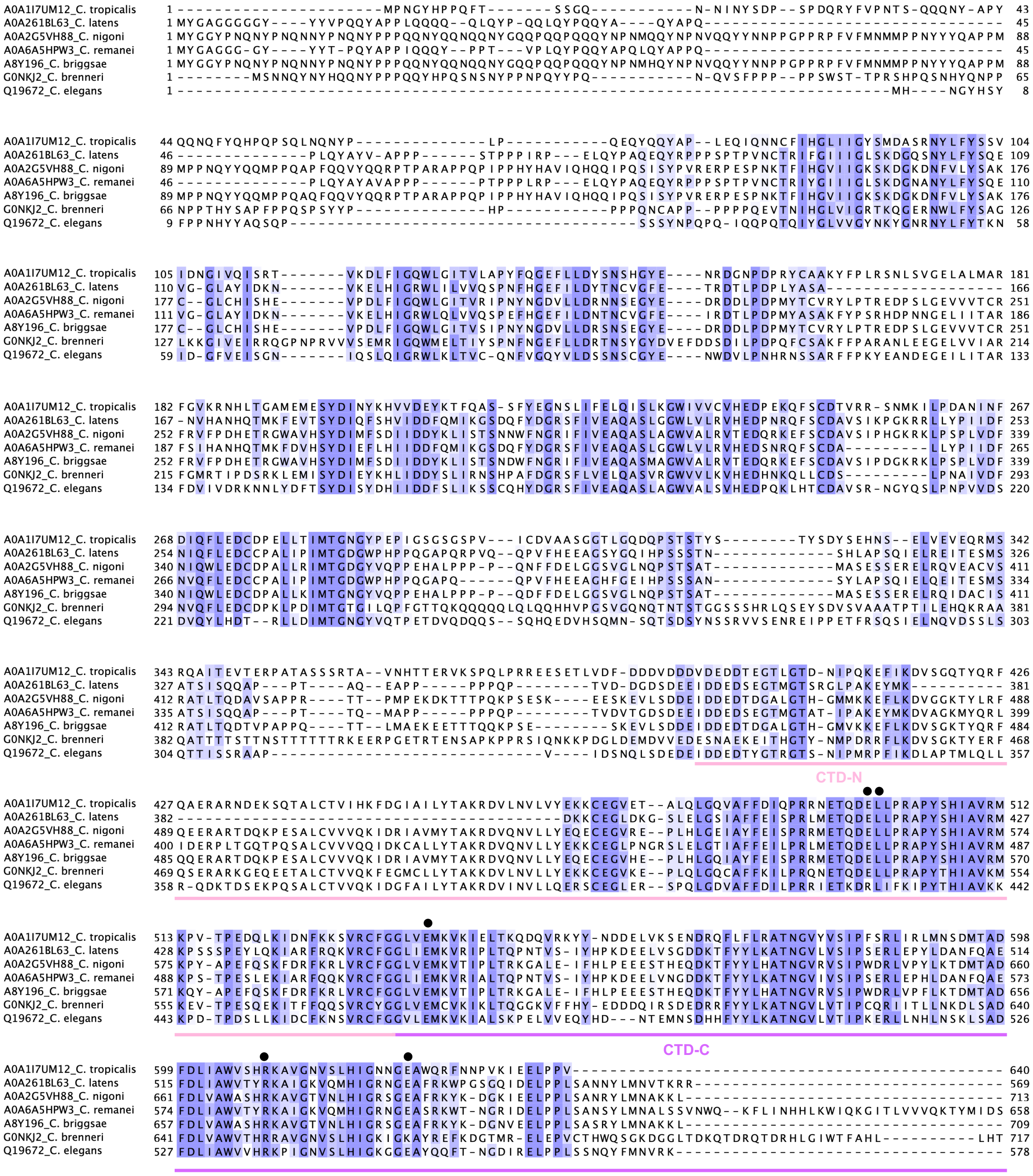

Supplementary Fig. 1: Multiple sequence alignment of different Caenorhabditis MUT-8 homologs. Residues are marked with shades of blue based on their conservation score. MUT-8 CTD subdomains are indicated with a light pink (CTD-N) or pink (CTD-C) line. MUT-8 residues involved in MUT-7 binding according to the crystal structure (PDB: 8Q66) are highlighted by black dots.

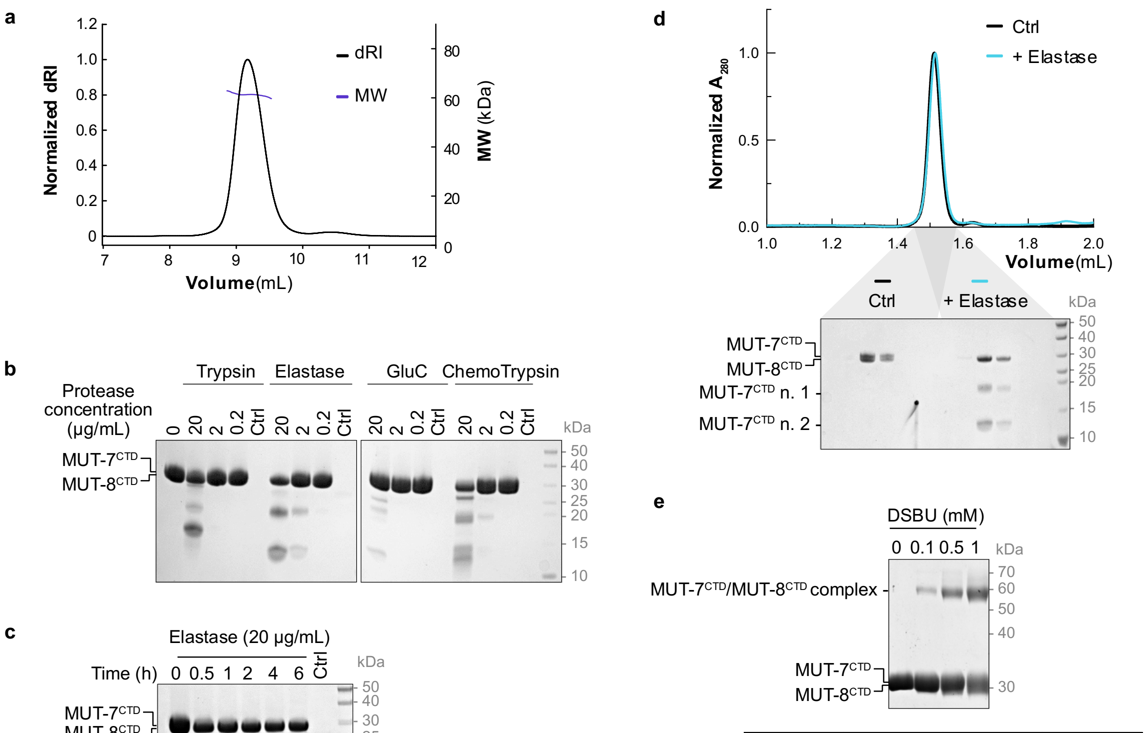

Protein #1	Site 1	Protein #2	Site 2
MUT-7	NH_2-N-Ter	MUT-7	K871
MUT-7	K701	MUT-8	T425
MUT-7	K698	MUT-8	K426
MUT-7	NH_2-N-Ter	MUT-7	K878

Supplementary Fig. 2: The MUT-7^{CTD}/MUT-8^{CTD} complex is a stable heterodimer.
a, SEC-Multi-Angle Light Scattering (SEC-MALS) analysis of the MUT-7^{CTD}/MUT-8^{CTD} complex. The sample was loaded on a Superdex 75 increase (10/300) column. Normalised differential Refractive Index (dRI) and calculated Molecular Weight (MW) are shown. **b**, Limited proteolysis of the purified MUT-7^{CTD}/MUT-8^{CTD} complex. MUT-7^{CTD}/MUT-8^{CTD} was incubated with decreasing concentrations of indicated proteases for 30 minutes on ice. The control (Ctrl) sample corresponds to the protease alone at the highest concentration. Reaction products were analysed by SDS-PAGE followed by Coomassie staining. MUT-7^{CTD} has a theoretical MW of 32 kDa and MUT-8^{CTD} of 29 kDa, being indistinguishable on the gel where they appear as a single band. **c**, Time-course of the limited proteolysis assay of the MUT-7^{CTD}/MUT-8^{CTD} complex. MUT-7^{CTD}/MUT-8^{CTD} was incubated with elastase (20 µg/mL) on ice for the indicated amounts of time. The control (Ctrl) sample corresponds to the elastase alone. A control reaction without protease was also performed. Reaction products were analysed by SDS-PAGE followed by Coomassie staining. **d**, Size exclusion chromatography analysis of the native (black, Ctrl) and elastase-treated (teal, + Elastase) MUT-7^{CTD}/MUT-8^{CTD} complex. The peak fractions were analysed by SDS-PAGE followed by Coomassie-staining. Upon elastase treatment, MUT-7^{CTD} (32 kDa) is cleaved into two fragments (MUT-7^{CTD} n.1 and MUT-7^{CTD} n.2), while MUT-8^{CTD} (29 kDa) remains intact. **e**, Crosslinking-MS analysis of the MUT-7^{CTD}/MUT-8^{CTD} complex. MUT-7^{CTD}/MUT-8^{CTD} was incubated with increasing concentrations of disuccinimidyldibutyric urea (DSBU). Samples were visualised by SDS-PAGE followed by Coomassie staining. The band at 60 kDa corresponding to the crosslinked MUT-7^{CTD}/MUT-8^{CTD} heterodimer was cut and analysed by MS. The table illustrates identified crosslinked residues.

a

	Foldseek Hits (TM-align)	Description	TM-score	RMSD (Å)
**MUT-7 CTD-N**	5tqj_A	Response regulator receiver protein (*B. phymatum*)	0.56	2.66
	6z2b_C	Toprim domain of RNase M5 (*G. stearothermophilus*)	0.60	3.20
**MUT-7 CTD-C**	NO HITS			
**MUT-8 CTD-N**	2a19_A	eIF2 alpha subunit (*S. cerevisiae*)	0.57	5.28
	7zhh_A	UnrCSD789 (*D. melanogaster*)	0.58	4.43
**MUT-8 CTD-C**	6cqo_H	Mitochondrial single-stranded DNA binding proteins Rim1 (*S. cerevisiae*)	0.61	3.03
	3k7u_C	MP18 RNA editing complex protein (*T. brucei*)	0.57	2.95

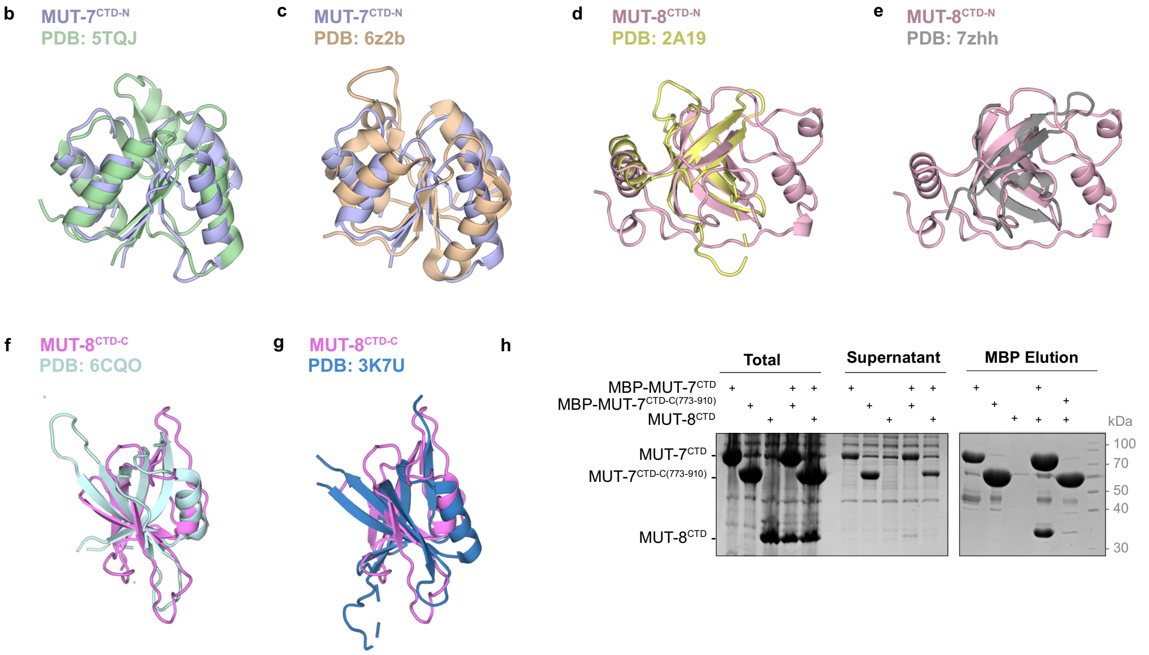

Supplementary Fig. 3: Structural analysis of MUT-7 CTD and MUT-8 CTD. a, Table with structures identified by Foldseek to be similar to MUT-7 CTD and MUT-8 CTD subdomains (CTD-N and CTD-C). TM-score and RMSD are reported. **b-g**, Structural alignments listed in (a). **b** and **c**, Structural alignment of MUT-7 CTD-N (light blue) with the response regulator receiver protein from *B. phymatum* (green) (51) (b) and the TOPRIM domain from *G. stearothermophilus* RNase M5 (wheat) (52) (c). **d** and **e**, Structural alignment of MUT-8 CTD-N (light pink) with the *S. cerevisiae* eukaryotic translation initiation factor 2 alpha subunit (yellow) (67) (d) and the *D. melanogaster* C-terminal cold shock domain of Upstream of N-Ras (grey) (68) (e). **f** and **g**, Structural alignment of MUT-8 CTD-C (magenta) with the *S. cerevisiae* mitochondrial single-stranded DNA binding protein Rim1 (cyan) (69) (f) and the *T. brucei* MP18 RNA editing complex protein (blue) (70) (g). **h**, Co-expression pulldown assay testing the interaction between two MBP-tagged MUT-7 constructs (MUT-7^{CTD} and MUT-7$^{CTD\text{-}C(773\text{-}910)}$) and MUT-8^{CTD}. MUT-7 constructs were co-expressed with MUT-8^{CTD} in *E. coli*. Expression of MUT-8^{CTD} alone was used as negative control. After lysis, the supernatant was incubated with amylose resin. Total lysate, supernatant and elution were analysed by SDS-PAGE followed by Coomassie staining.

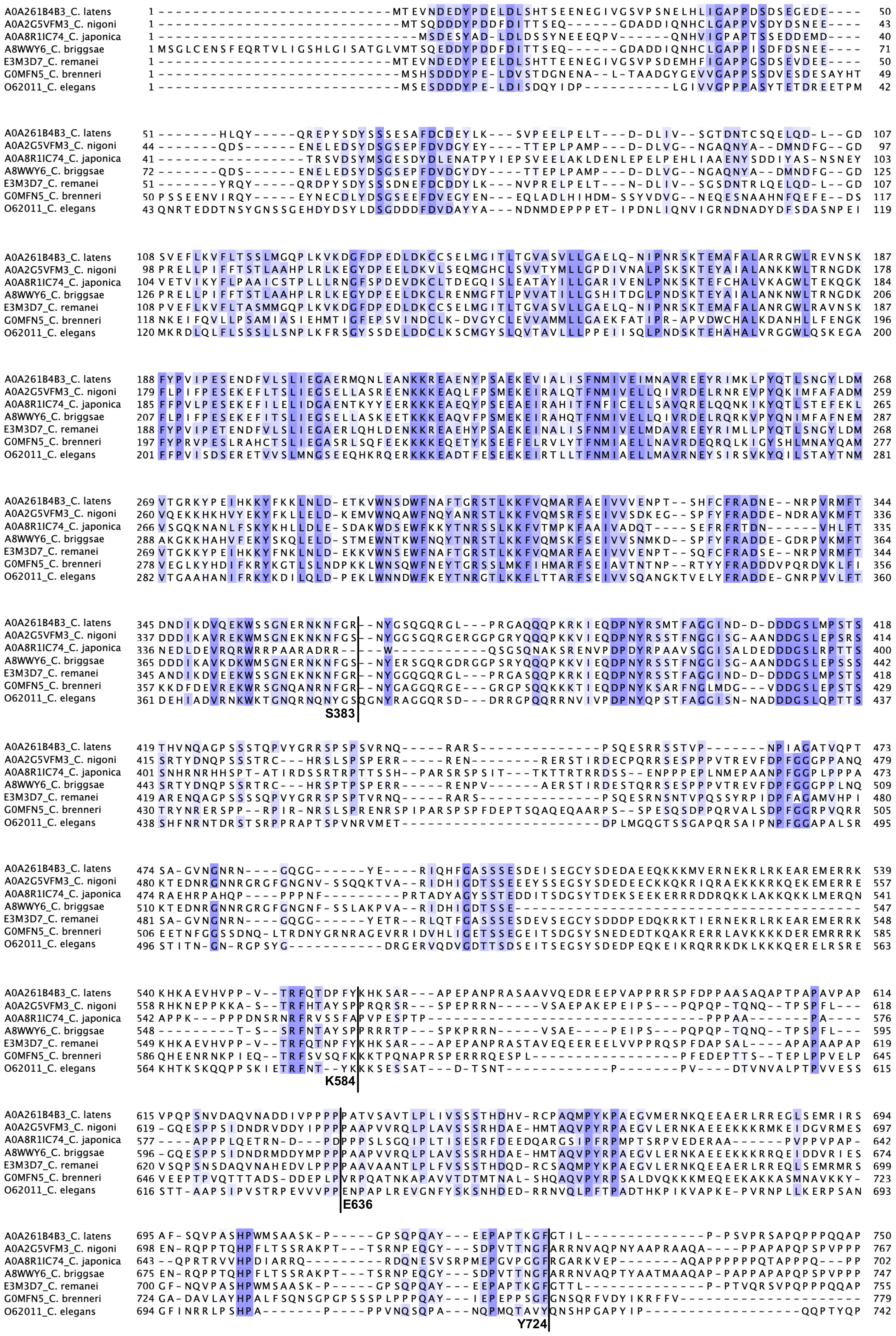
A0A261B4B3_C. latens
A0A2G5VFM3_C. nigoni
A0A8R1IC74_C. japonica
A8WWY6_C. briggsae
E3M3D7_C. remanei
G0MFN5_C. brenneri
O62011_C. elegans
S383
K584
E636
Y724

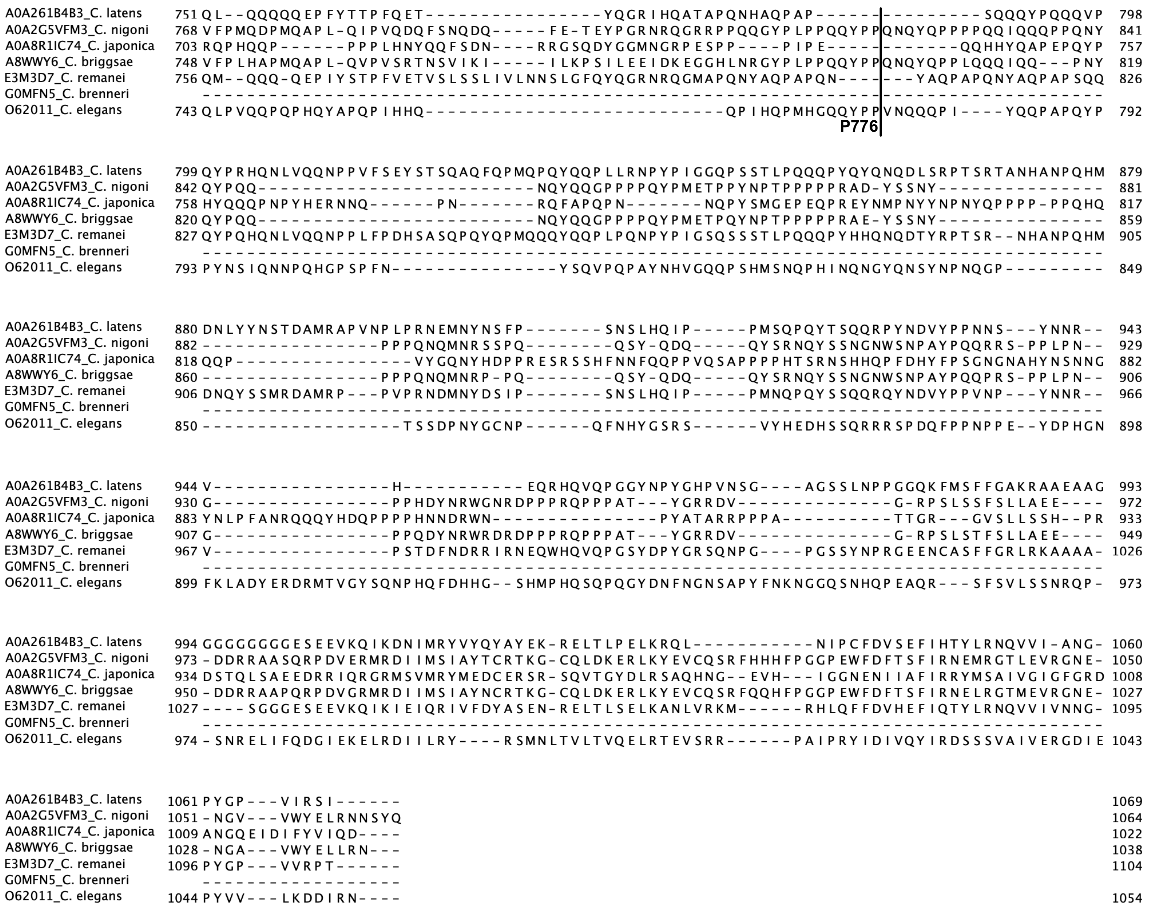

Supplementary Fig. 4: Multiple sequence alignment of different Caenorhabditis MUT-16 homologs. Residues are marked with shades of blue based on their conservation score. Boundaries of MUT-16 constructs mentioned in the text are highlighted.

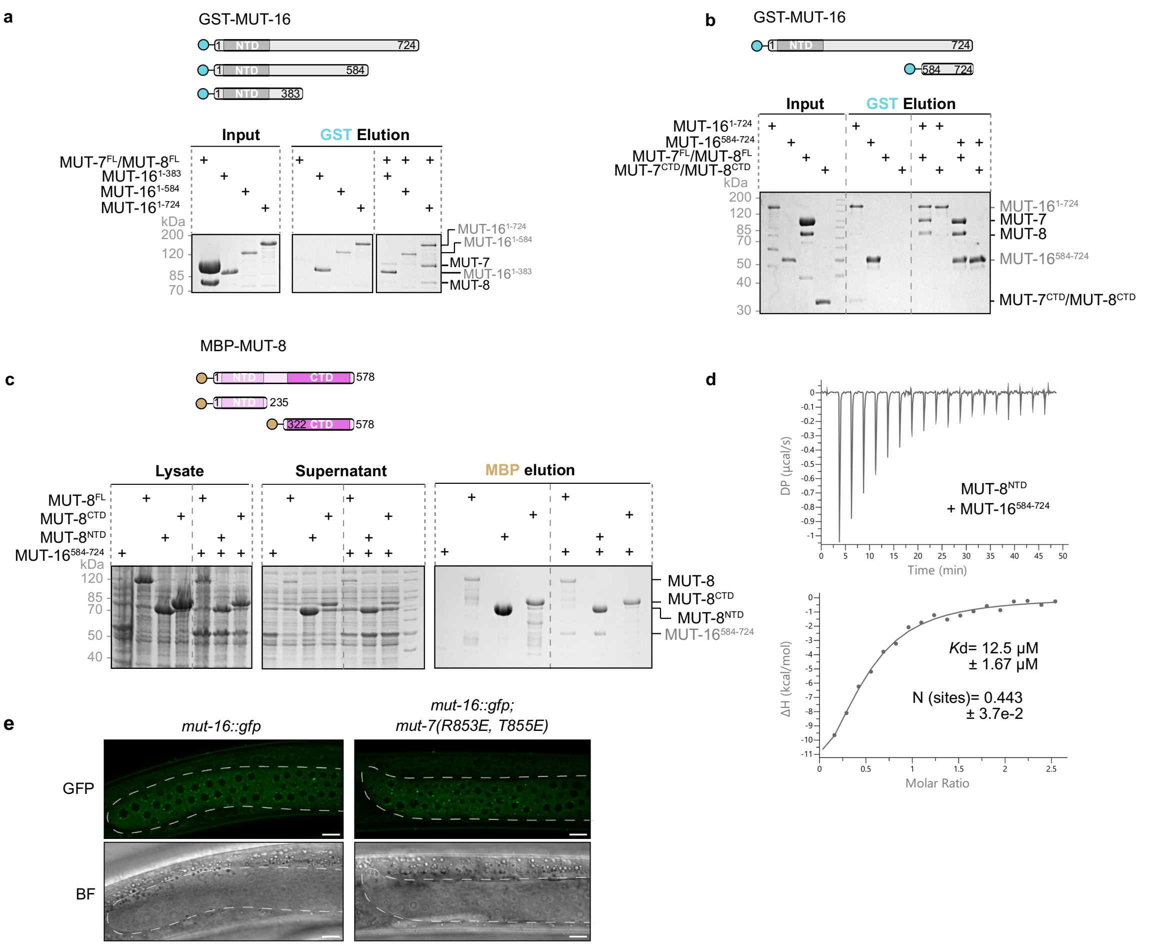

Supplementary Fig. 5: MUT-8 directly interacts with MUT-16. a, GST pulldown assessing the binding of three different GST-tagged MUT-16 constructs (MUT-16$^{1\text{-}724}$, MUT-16$^{1\text{-}584}$ and MUT-16$^{1\text{-}383}$) to the untagged MUT-7/MUT-8 complex. The cartoon illustrates the three MUT-16 constructs used as bait. Purified proteins were incubated with glutathione-coupled beads. Incubation of untagged MUT-7/MUT-8 with glutathione-coupled beads was used as negative control. Input and elution fractions were analysed by SDS-PAGE followed by Coomassie staining. **b**, GST pulldown assessing the binding of two GST-tagged MUT-16 constructs (MUT-16$^{1\text{-}724}$ and MUT-16$^{584\text{-}724}$) to the untagged MUT-7/MUT-8 and MUT-7^{CTD}/MUT-8^{CTD} complexes. The cartoon illustrates the two MUT-16 constructs used as bait. Purified proteins were incubated with glutathione-coupled beads. Incubation of untagged MUT-7/MUT-8 and MUT-7^{CTD}/MUT-8^{CTD} complexes with glutathione-coupled beads was used as negative control. Input and elution fractions were analysed by SDS-PAGE followed by Coomassie staining. **c**, Co-expression pulldown assay testing the interaction between MBP-tagged MUT-8 constructs (MUT-8, MUT-8^{NTD} and MUT-8^{CTD}) and GST-tagged MUT-16$^{584\text{-}724}$. The cartoon illustrates the three MUT-8 constructs used as bait. MUT-8 constructs were co-expressed with MUT-16$^{584\text{-}724}$ in *E. coli*. Expression of GST-tagged MUT-16$^{584\text{-}724}$ alone was used as negative control. After lysis, the supernatant was incubated with amylose resin. Total lysate, supernatant and elution are analysed by SDS-PAGE followed by Coomassie staining. **d**, Isothermal titration calorimetry (ITC) experiment analysing the interaction between MUT-8^{NTD} and MUT-16$^{584\text{-}724}$. One representative of three ITC experiments is shown. The calculated dissociation constant (K_d) and the number of binding sites (N) are the mean of three experiments, and the error bars correspond to ± SD. We

note that the stoichiometry determined by ITC deviates from the expected 1 to 1 ratio. **e**, Live worm imaging showing MUT-16-GFP localisation in WT and *mut-7(R853E, T855E)* germline. The mitotic part of the young adult gonad is shown. A representative image from a total of three analysed animals is shown. BF: Bright Field. Scale bars: 10 µm.

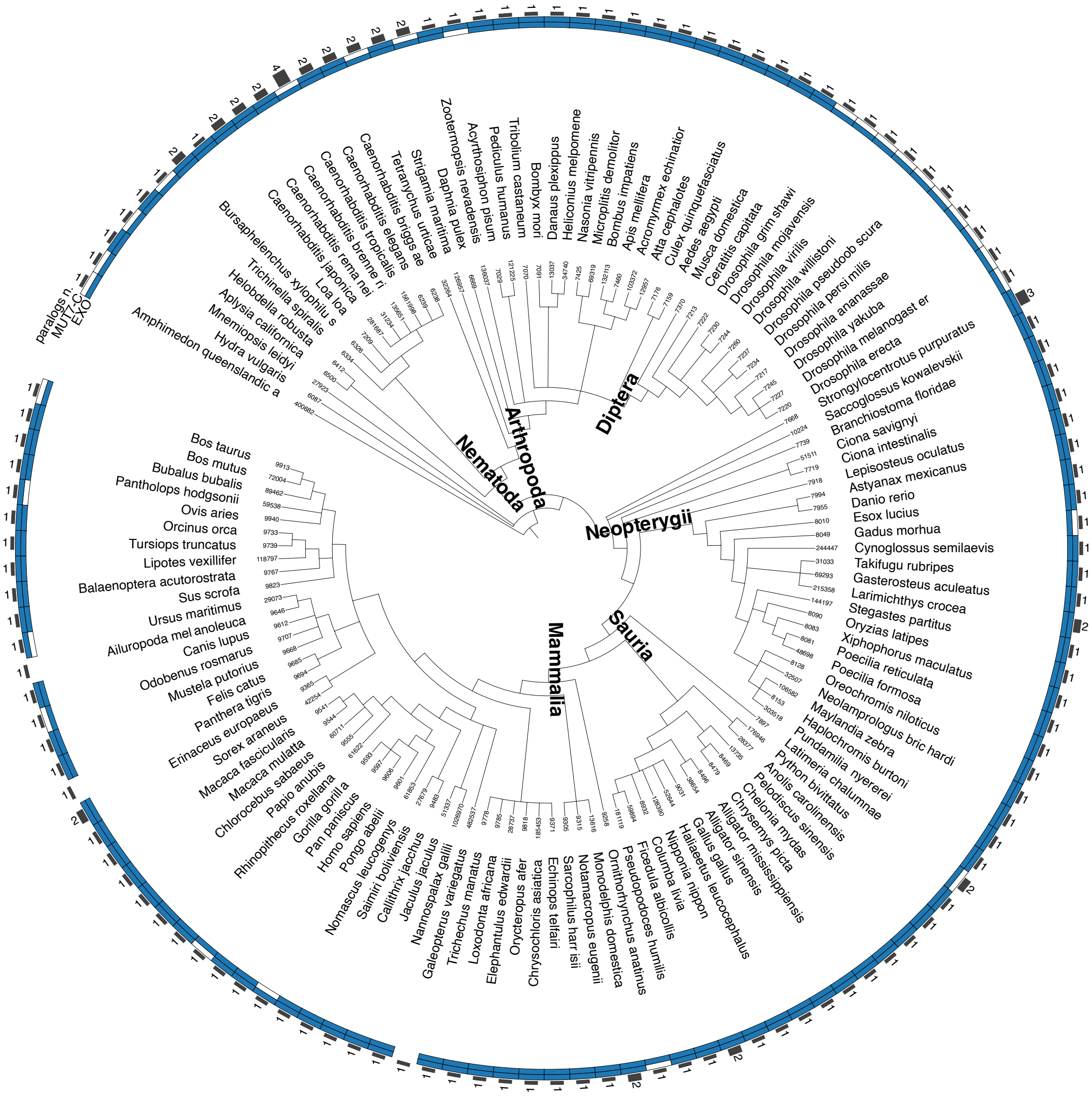

Supplementary Fig. 6: Phylogenetic species tree illustrating the conservation of MUT-7 and its CTD across metazoan. The tree is based on the EggNOG non-supervised orthologous group ENOG503BGHH (taxonomy level metazoan). The presence of MUT-7 exoribonuclease domain (PF01612) and its C-terminal domain (PF01927) in each species is marked in blue. The number of predicted homologs per species is also indicated.

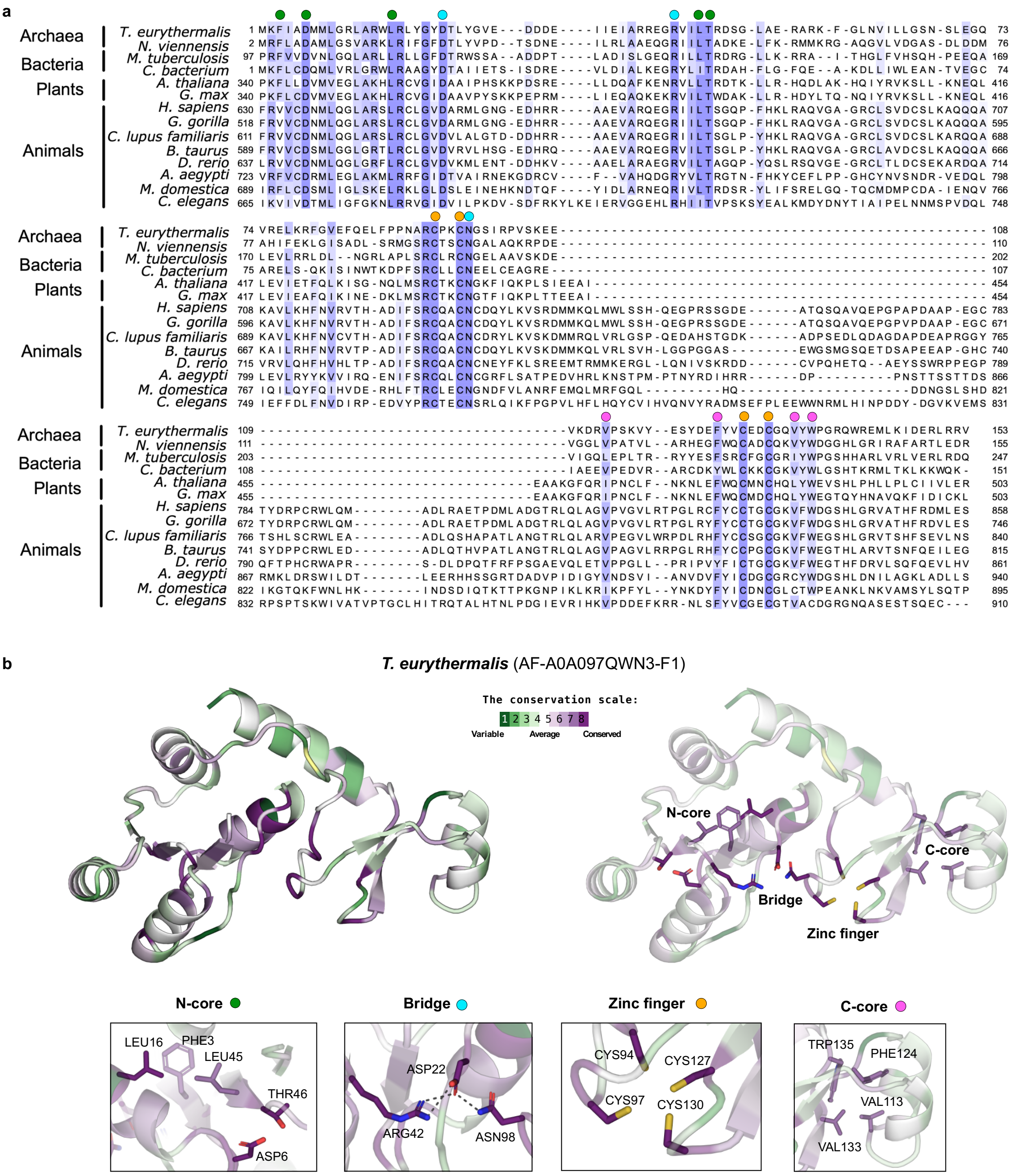

Supplementary Fig. 7: MUT7-C domain conservation. a, Multiple sequence alignment of prokaryotic and eukaryotic MUT7-C domains. The conserved residues described in (b) are highlighted in green (for the N-core), cyan (for the Bridge), orange (for the Zinc finger) and magenta (for the C-core). **b**, Cartoon representation of the MUT7-C domain-containing protein A0A097QWN3 from the archaeon *Thermococcus eurythermalis* (AlphaFold prediction) coloured according to conservation. Conservation scores were calculated using ConSurf, with the sequences shown in (a). The gradient from green to purple indicates increasingly conserved residues. MUT-7^{CTD-N} contains a cluster of hydrophobic and charged residues that build the N-terminal core (N-core). The MUT-7^{CTD-C} is characterised by a zinc finger formed by the four invariant cysteine residues (zinc finger) and a small core of hydrophobic and aromatic residues that flank the two C-terminal Cysteines of the zinc-ribbon (C-core). MUT-7^{CTD-C} is connected to MUT-7^{CTD-N} via a bridge element (Bridge) that is formed by the MUT-7^{CTD-C} Asn98 flanking the first two zinc-ribbon Cysteines and Arg42 and Asp22 in MUT-7^{CTD-N}.

a

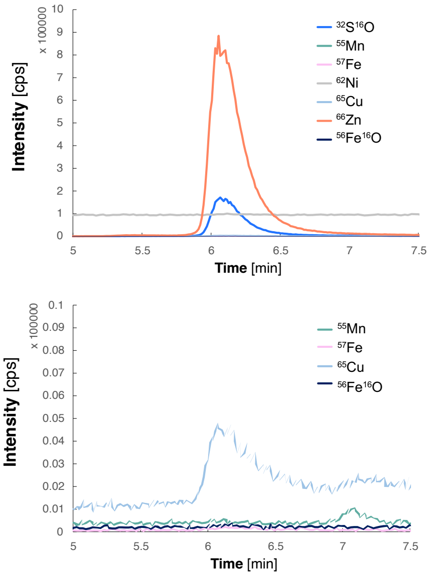

b

Sample Name	$^{32}S^{16}O$ peak (counts)	^{66}Zn peak (counts)	S/Zn intensity ratio	Stoichiometry[1] (mol Zn/ mol Protein)
EXD3^{CTD} Replicate_1	2879558	15433709	0.18658	1.02
EXD3^{CTD} Replicate_2	2366140	13127137	0.18025	1.06
EXD3^{CTD} Replicate_3	2311390	12799837	0.18058	1.05
**Average**				**1.04**
**RSD (n=3)**				**2%**

[1]A total combined uncertainty of 10% of the measured stoichiometry is estimated

c

	150 mM NaCl			
**Fit values**	**MUT-7^{FL}**	**MUT-7$^{NTD\ EXO}$**	**MUT-7^{CTD}**	**MUT-7^{CTD}/MUT-8^{CTD}**
**B_{max}** (mP)	213.2	46.01	25032	82803
**h**	1.195	1.491	0.4737	0.6777
**K_d** (µM)	2.325	18.69	Unstable	Unstable

d

	250 mM NaCl		
**Fit values**	**EXD3^{FL}**	**EXD3$^{NTD-EXO}$**	**EXD3^{CTD}**
**B_{max}** (mP)	215	149.9	47834
**h**	1.029	1.1	0.8825
**K_d** (µM)	1.862	11.15	Unstable

e

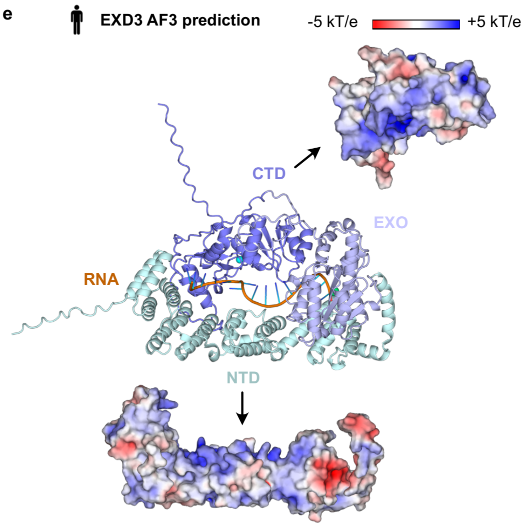

Supplementary Fig. 8: MUT-7 and EXD3 CTDs contribute to RNA binding. a and **b**, SEC-ICP-MS of human EXD3 CTD. **a**, Chromatograms showing the count per second (cps) obtained over time for different species. A zoom-in is shown in the lower panel. Data are representative of three technical replicates. **b**, Table summarizing the count per second (cps) values obtained for ^{66}Zn and $^{32}S^{16}O$ in each of the three replicates. The intensity ratio between the ^{66}Zn and the $^{32}S^{16}O$ signals was calculated. Comparison of this ratio with the value obtained for the calibrant (Bovine Cu/Zn-superoxide dismutase) allowed the determination of the number of Zinc ions bound to each EXD-3 molecule. **c**, Fit values for maximum specific binding value (B_{max}), hill coefficient (h) and dissociation constant (K_d) of the experiment testing MUT-7 affinity for a 16-mer RNA (see Fig. 7a). **d**, Fit values for maximum specific binding value (B_{max}), hill coefficient (h) and dissociation constant (K_d) of the experiment testing EXD3 affinity for a 16-mer RNA (see Fig. 7b). **e**, AlphaFold3 prediction of EXD3 bound to a 16-mer ssRNA, Mn^{2+} and Zn^{2+}. pTM=0.56, ipTM=0.43. The EXD3 domains are in shades of blue, RNA in orange, Zn^{2+} in cyan, Mn^{2+} in green. The electrostatic surface potential of the NTD and CTD was calculated by Adaptive Poisson-Boltzmann Solver from −5 kT/e (red) to +5 kT/e (blue).

Supplementary Tables

Table S1: Recombinant proteins and purification strategies used in this study.
"/" indicates that the two proteins were co-expressed and purified as a complex. (N) and (C) indicate the location of the tag at either the N- or C-terminus. His10 or His6 indicate the presence of 10 or 6 Histidine residues. MBP = Maltose Binding Protein, Trx = Thioredoxin, GST = Glutathione S-transferase, 3C = 3C cleavage site (LEVLFQ/GP), SEC = Size Exclusion Chromatography, SEC-MALS = Size Exclusion Chromatography (SEC) coupled to multi-angle light scattering (MALS), CD= Circular dichroism.

Name	Residues	Tags	Purification Steps	Plasmid #	Use
MUT-7^{FL}	1-910	(N) His10-MBP-3C	MBPTrap, 3C, Heparin, SEC	Ce255	Ribonuclease assay
MUT-7^{FL}	1-910	(N) MBP-3C, (C) His10	HISTrap, 3C, Heparin, SEC	Ce409	Ribonuclease assay, Anisotropy
MUT-7$^{NTD-EXO}$	1-625	(N) His10-MBP-3C	HISTrap, 3C, HISTrap, Heparin, SEC	Ce333	Ribonuclease assay
MUT-7^{CTD}	633-910	(N) His10-MBP-3C	HISTrap, 3C, HISTrap, Anion exchange, SEC	Ce285	Ribonuclease assay, Anisotropy
MUT-7^{FL} /MUT-8^{FL}	1-910 /1-578	(N) His10-MBP-3C /(N) His6-Trx-3C	MBPTrap, 3C, Heparin, Anion exchange, SEC	Ce255 /Ce15	Ribonuclease assay, Phase separation, SEC
MUT-7^{D435A} /MUT-8^{CTD}	1-910 /322-578	(N) His10-MBP-3C /(N) His10-MBP-3C	MBPTrap, 3C, Heparin, SEC	Ce256 /Ce286	Ribonuclease assay
MUT-7^{CTD} /MUT-8^{CTD}	633-910 /322-578	(N) His10-MBP-3C /(N) His10-MBP-3C	HISTrap, 3C, HISTrap, Anion exchange, SEC	Ce285 /Ce286	Pulldown, Proteolysis, Crosslinking, SEC-MALS, Anisotropy
MUT-7$^{CTD(633-899)}$ /MUT-8$^{CTD(322-567)}$	633-899 /322-567	(N) His10-MBP-3C /(N) His10-MBP-3C	HISTrap, 3C, HISTrap, Anion exchange, SEC	Ce373 /Ce374	Crystal screenings and structure
MUT-8^{NTD}	1-235	(N) His10-MBP-3C	HISTrap, 3C, Anion exchange, SEC	Ce339	CD, SEC
MUT-16$^{584-724}$ /MUT-8^{NTD}	584-724 /1-235	(N) His6-GST-3C /(N) His10-MBP-3C	HISTrap, 3C, HISTrap, SEC	Ce348 /Ce339	Phase separation, CD, SEC
MUT-16$^{1-724}$	1-724	(N) GST-3C, (C) His10	HISTrap, Anion exchange, SEC	Ce221	Pulldown
MUT-16$^{1-584}$	1-584	(N) GST-3C, (C) His10	HISTrap, Anion exchange, SEC	Ce220	Pulldown
MUT-16$^{1-383}$	1-383	(N) GST-3C, (C) His10	HISTrap, Anion exchange, SEC	Ce219	Pulldown
MUT-16$^{584-724}$	584-724	(N) GST-3C, (C) His10	HISTrap, 3C, Cation exchange	Ce298	Phase separation, CD
MUT-16$^{584-724}$	584-724	(N) GST-3C, (C) His10	HISTrap, Anion	Ce298	Pulldown

			exchange, SEC		
EXD-3^{FL}	1-876	(N) His10-MBP-3C	HISTrap, Heparin, 3C, HISTrap, SEC	Hs87	Ribonuclease assay
EXD-3$^{NTD\text{-}EXO}$	1-582	(N) His10-MBP-3C	HISTrap, 3C, HISTrap, Heparin, SEC	Hs88	Ribonuclease assay
EXD-3^{CTD}	624-876	(N) His10-MBP-3C	HISTrap, 3C, Heparin, SEC	Hs89	Anisotropy, ribonuclease assay, SEC-ICP-MS
EXD-3$^{FL(D399A)}$	1-876	(N) His10-MBP-3C	HISTrap, 3C, Heparin, SEC	Hs90	Anisotropy
EXD-3$^{NTD\text{-}EXO(D399A)}$	1-582	(N) His10-MBP-3C	HISTrap, 3C, Heparin, SEC	Hs91	Anisotropy

Table S2: Constructs used for co-expression pulldown assays.
Constructs only used for co-expression pulldown assays and not for protein purification are highlighted in bold. (N) and (C) indicate that the tag is N-terminally or C-terminally fused to the protein of interest, respectively. His10 or His6 indicate the presence of 10 or 6 Histidine residues, respectively. MBP= Maltose Binding Protein, Trx= Thioredoxin, GST= Glutathione S-transferase, 3C= 3C cleavage site (LEVLFQ/GP).

**Name**	**Residues**	**Tags**	**Plasmid #**
MUT-7^{FL}	1-910	(N) His10-MBP-3C	Ce255
MUT-7$^{NTD\text{-}EXO}$	1-625	(N) His10-MBP-3C	Ce333
MUT-7^{CTD}	633-910	(N) His10-MBP-3C	Ce285
**MUT-7$^{CTD(R853E, T855E)}$**	**633-910**	**(N) His10-MBP-3C**	**Ce302**
**MUT-7$^{CTD\text{-}C(773\text{-}910)}$**	**773-910**	**(N) His10-MBP-3C**	**Ce349**
MUT-8^{FL}	1-578	(N) His6-Trx-3C	Ce15
**MUT-8^{FL}**	**1-578**	**(N) His10-MBP-3C**	**Ce338**
MUT-8^{NTD}	1-235	(N) His10-MBP-3C	Ce339
**MUT-8^{CTD}**	**322-578**	**(N) His10-3C**	**Ce299**
MUT-16$^{1\text{-}724}$	1-724	(N) GST-3C, (C) His10	Ce221
MUT-16$^{1\text{-}584}$	1-584	(N) GST-3C, (C) His10	Ce220
MUT-16$^{1\text{-}383}$	1-383	(N) GST-3C, (C) His10	Ce219
MUT-16$^{584\text{-}724}$	584-724	(N) GST-3C, (C) His10	Ce298
HsEXD-3^{CTD}	624-876	(N) His10-MBP-3C	Hs89
**DrEXD-3^{CTD}**	**631-861**	**(N) His10-MBP-3C**	**Dr05**

Table S3: Data collection and refinement statistics

Rmsd: Root mean square deviation.

https://doi.org/10.2210/pdb8Q66/pdb

Dataset	**MUT-7^{CTD}/MUT-8^{CTD}**
PDB	8Q66
Space group	C 1 2 1
Cell dimensions	
a, b, c (Å)	145.09, 59.05, 88.16
α, β, γ (°)	90.0, 119.8, 90.0
**Data Collection**	
Wavelength (Å)	0.87
Resolution range	67.91 – 2.03 (2.10 – 2.03)
No. of reflections	146,924 (15006)
No. of unique reflection	42,083 (4152)
R_{merge} (%)	8.9 (127.2)
R_{pim} (%)	5.6 (100)
$I / \sigma I$	9.1 (0.8)
Completeness (%)	99.7 (99.6)
Multiplicity	3.5 (3.6)
$CC_{1/2}$	1 (0.41)
**Refinement**	
Resolution range	62.95 – 2.03 (2.10 – 2.03)
Reflections used in refinement	41,976 (4145)
R_{work} / R_{free} (%)	18.8 / 22.1
Wilson B-factor (Å^2)	40.55
Average B-factors (Å^2)	49.52
**No. of atoms**	
Proteins	4168
Ligands	31
Solvent	250
**Stereochemistry**	
Rmsd bond lengths (Å)	0.0103
Rmsd bond angles (°)	0.56
Ramachandran favored (%)	97.8
Ramachandran outliers (%)	0.2

Table S4: List of *C. elegans* strains used in this study.
RNAi: RNA interference; IF: Immunofluorescence; WB: Western Blot

Strain	Genotype	Use	Reference
N2	*C. elegans* wild isolate var Bristol	RNAi, IF, WB	Caenorhabditis Genetics Center
RFK502	*mut-7 (pk204) III*	RNAi	(22)
RFK1714	*mut-7(xf367[mut-7(R853E, T855E)]) III*	RNAi	This study
RFK1713	*mut-16(cmp3[mut-16::gfp::flag + loxP]) I*	Live imaging	(71)
RFK1716	*mut-16(cmp3[mut-16::gfp::flag + loxP]) I;*<br>*mut-7(xf367[mut-7(R853E, T855E)]) III*	Live imaging	This study
RFK1754	*mut-7(xf379[HA::mut-7])III*	IF, WB	This study
RFK1755	*mut-7(xf380[HA::mut-7(R853E, T855E)])III*	IF, WB	This study

Supplementary references

62. Rappsilber,J., Mann,M. and Ishihama,Y. (2007) Protocol for micro-purification, enrichment, pre-fractionation and storage of peptides for proteomics using StageTips. *Nat. Protoc.*, **2**, 1896–1906.
63. Tyanova,S., Temu,T. and Cox,J. (2016) The MaxQuant computational platform for mass spectrometry-based shotgun proteomics. *Nat. Protoc.*, **11**, 2301–2319.
64. Iacobucci,C., Götze,M., Ihling,C.H., Piotrowski,C., Arlt,C., Schäfer,M., Hage,C., Schmidt,R. and Sinz,A. (2018) A cross-linking/mass spectrometry workflow based on MS-cleavable cross-linkers and the MeroX software for studying protein structures and protein-protein interactions. *Nat. Protoc.*, **13**, 2864–2889.
65. Combe,C.W., Fischer,L. and Rappsilber,J. (2015) xiNET: cross-link network maps with residue resolution. *Mol. Cell. Proteomics MCP*, **14**, 1137–1147.
66. Hann,S., Koellensperger,G., Obinger,C., Furtmüller,P.G. and Stingeder,G. (2004) SEC-ICP-DRCMS and SEC-ICP-SFMS for determination of metal–sulfur ratios in metalloproteins. *J. Anal. At. Spectrom.*, **19**, 74–79.
67. Dar,A.C., Dever,T.E. and Sicheri,F. (2005) Higher-Order Substrate Recognition of eIF2α by the RNA-Dependent Protein Kinase PKR. *Cell*, **122**, 887–900.
68. Hollmann,N.M., Jagtap,P.K.A., Linse,J.-B., Ullmann,P., Payr,M., Murciano,B., Simon,B., Hub,J.S. and Hennig,J. (2023) Upstream of N-Ras C-terminal cold shock domains mediate poly(A) specificity in a novel RNA recognition mode and bind poly(A) binding protein. *Nucleic Acids Res.*, **51**, 1895–1913.
69. Singh,S.P., Kukshal,V., De Bona,P., Antony,E. and Galletto,R. (2018) The mitochondrial single-stranded DNA binding protein from S. cerevisiae, Rim1, does not form stable homo-tetramers and binds DNA as a dimer of dimers. *Nucleic Acids Res.*, **46**, 7193–7205.
70. Wu,M., Park,Y.-J., Pardon,E., Turley,S., Hayhurst,A., Deng,J., Steyaert,J. and Hol,W.G.J. (2011) Structures of a key interaction protein from the Trypanosoma brucei editosome in complex with single domain antibodies. *J. Struct. Biol.*, **174**, 124–136.
71. Wan,G., Fields,B.D., Spracklin,G., Shukla,A., Phillips,C.M. and Kennedy,S. (2018) Spatiotemporal regulation of liquid-like condensates in epigenetic inheritance. *Nature*, **557**, 679–683.
